# Melanoma cells undergo aggressive coalescence in a 3D Matrigel model that is repressed by anti-CD44

**DOI:** 10.1371/journal.pone.0173400

**Published:** 2017-03-06

**Authors:** Deborah Wessels, Daniel F. Lusche, Edward Voss, Spencer Kuhl, Emma C. Buchele, Michael R. Klemme, Kanoe B. Russell, Joseph Ambrose, Benjamin A. Soll, Aaron Bossler, Mohammed Milhem, Charles Goldman, David R. Soll

**Affiliations:** 1 Developmental Studies Hybridoma Bank, Department of Biology, University of Iowa, Iowa City, IA United States of America; 2 Department of Molecular Pathology, University of Iowa Hospitals and Clinics, Iowa City, IA United States of America; 3 Department of Internal Medicine, University of Iowa Hospitals and Clinics, Iowa City, IA United States of America; 4 Mercy Hospital System of Des Moines, Des Moines, IA United States of America; University of Alabama at Birmingham, UNITED STATES

## Abstract

Using unique computer-assisted 3D reconstruction software, it was previously demonstrated that tumorigenic cell lines derived from breast tumors, when seeded in a 3D Matrigel model, grew as clonal aggregates which, after approximately 100 hours, underwent coalescence mediated by specialized cells, eventually forming a highly structured large spheroid. Non-tumorigenic cells did not undergo coalescence. Because histological sections of melanomas forming in patients suggest that melanoma cells migrate and coalesce to form tumors, we tested whether they also underwent coalescence in a 3D Matrigel model. Melanoma cells exiting fragments of three independent melanomas or from secondary cultures derived from them, and cells from the melanoma line HTB-66, all underwent coalescence mediated by specialized cells in the 3D model. Normal melanocytes did not. However, coalescence of melanoma cells differed from that of breast-derived tumorigenic cell lines in that they 1) coalesced immediately, 2) underwent coalescence as individual cells as well as aggregates, 3) underwent coalescence far faster and 4) ultimately formed long, flat, fenestrated aggregates that were extremely dynamic. A screen of 51 purified monoclonal antibodies (mAbs) targeting cell surface-associated molecules revealed that two mAbs, anti-beta 1 integrin/(CD29) and anti-CD44, blocked melanoma cell coalescence. They also blocked coalescence of tumorigenic cells derived from a breast tumor. These results add weight to the commonality of coalescence as a characteristic of tumorigenic cells, as well as the usefulness of the 3D Matrigel model and software for both investigating the mechanisms regulating tumorigenesis and screening for potential anti-tumorigenesis mAbs.

## Introduction

Cancer cells exhibit a number of characteristics not normally exhibited by non-cancer cells. These can include resistance to signals that inhibit cell multiplication [1–4], growth factor independence [5, 6], a decrease in programmed cell death [7–9], self-signaling to stimulate cell multiplication [10–13], invasiveness and metastasis [14], tumorigenesis in animal models [15–17], and a number of additional characteristics [1, 2]. Recently, we demonstrated that tumorigenic cell lines derived from breast tumors, but not non-tumorigenic cell lines, also possess the capacity to generate large cell aggregates in a 3D Matrigel model through coalescence of clonal aggregates formed through the multiplication of single cells seeded in the gel [18, 19]. The process of coalescence of aggregates occurs after an extended growth period and is mediated by specialized cells that recruit other cells from the aggregates to form cables between aggregates that contract, actively moving smaller into larger aggregates [18, 19]. Eventually, through continued coalescence the majority of cells in a 3D field coalesce into single large aggregate that then differentiates into a highly structured hollow sphere of cells. The process of coalescence has been interpreted to mimic or reflect some aspects of tumorigenesis *in vivo* [18, 19] most notably coalescence in “field cancerization” [20]. Field cancerization was first articulated by Slaughter et al. (1953) [20], and was subsequently noted in a variety of cancers [21–30]. It was suggested that multiple tumorigenic foci within a cancerized field coalesce and that coalescence contributes to tumor growth as well as tumor heterogeneity [20].

**Fig 1:**
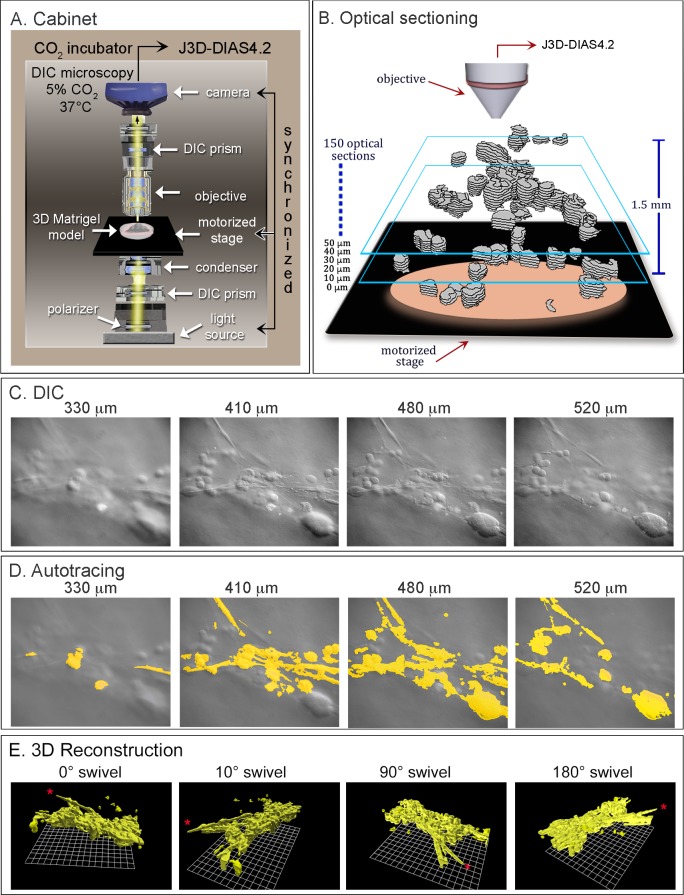
4D reconstructions are generated with a customized cabinet and J3D-DIAS4.2 software. A. The 3D platform included an upright light microscope supporting the 3D Matrigel preparation seeded with cells, a computer-synchronized mechanical stage, camera and light source for optical sectioning, and DIC optics. This system is housed in a 5% CO_2_ incubator and maintained at 37°C. B. One hundred and fifty optical sections were collected in a 30 second period, as cartooned in this panel, and this sectioning process repeated every 10 minutes. Optical sections are drawn in grey. Blue boxes denote different depths. C. DIC optical sections obtained at four depths during one series of sectioning. D. Computer-assisted autotracing of the optical sections in C. E. 3D reconstruction by J3D-DIAS4.2 of an aggregate formed by coalescence, viewed from four angles.

J3D-DIAS 4.2 [31, 32], the 4D computer-assisted system developed in our laboratory and used to reconstruct and analyze coalescence, relies on differential interference contrast (DIC) microscopy. DIC microscopy allows optical sectioning of living preparations at very short time intervals, produces negligible heat [33–35] and does not rely on staining or fluorescence, thus circumventing phototoxicity problems [35–37]. In the 3D model, cold liquid Matrigel is seeded with single cells and allowed to gel, resulting in independent cells dispersed throughout the gel. A 3D region of the live preparation is then optically sectioned in a 45 second period, producing a stack of 150 DIC optical sections through 1500 μm (Fig 1). This process is repeated every 10 minutes for as long as the experiment requires (up to one month, if desired). The J3D-DIAS 4.2 software then reconstructs the cells and aggregates from the optical sections every 10 minutes. Because the reconstructed cells and aggregates are converted into mathematical models, the software provides not only unique dynamic 3D videos of aggregate growth and coalescence, but also calculations of a variety of 3D aggregation, motility and contour parameters over time [31, 32].

The 3D Matrigel model and evolving 4D reconstruction and motion analysis software [31, 32] were initially used to analyze tumorigenic cell lines derived from breast tumors [18, 19]. However, histological descriptions of melanoma development suggested that the model may be more relevant to the formation of melanomas *in vivo*. Histological studies suggested that individual melanoma cells first undergo pagetoid spreading through the skin [38, 39], especially in superficial spreading malignant melanomas [40]. Images of spreading cells have been referred to as “buckshot” in pathological profiles [40–45]. Eventually, “nests” of malignant cells form, which are architecturally round to irregular [40–45]. Most importantly, these nests can coalesce [44]. Cellular bridges or cables, can be observed between nests in histological sections [44]. Eventually nests can spread horizontally along the skin, as well as vertically towards the dermis [40, 41]. These observations suggest that aggregate coalescence and the mediation of coalescence between aggregates by cables of specialized cells, which we demonstrated in 3D Matrigel models of breast tumor-derived cell lines *in vitro* [18, 19], may indeed occur in the progression of melanomas *in vivo*. Moreover, there are reasons to believe that melanoma cells may be more aggressive in behavior than breast cancer cells in the process of metastasis. A major comparison by the National Cancer Institute between 2006 and 2012 between breast cancer and melanoma patients of five-year survival rates, revealed dramatically lower rates of survival for melanoma patients, (http://seer.cancer.gov/statfacts/html/breast/html). In addition, Gupta et al. (2005) [46] showed that overexpression of the oncogene Ras in normal melanocytes induced tumorigenesis and metastasis, whereas overexpression of Ras in normal epithelial cells resulted in tumorigenesis, but not metastasis. These results suggest that melanocytes already possess a propensity greater than breast cells to become metastatic.

Here we have tested whether cells from three fresh melanomas and an established melanoma cell line undergo coalescence between aggregates formed by cell multiplication, whether bridges of specialized cells mediate coalescence, and whether the developmental program for melanoma coalescence is similar or different from that of breast tumor-derived cells in the 3D Matrigel model. It is demonstrated that melanoma cells from fresh tumors or an established melanoma cell line indeed undergo aggregate coalescence and that coalescence of aggregates is mediated by the formation of cell bridges formed by behaviorally distinct cells, as is the case for tumorigenic breast cancer-derived cell bridges [18, 19]. However, the results also demonstrate behavioral characteristics specific to melanoma cells, both fresh cells from tumors secondary melanoma cell cultures and an established melanoma cell line. It has expanded our definition of coalescence to include not only the active fusion of aggregates facilitated by specialized cells, to active cell-cell aggregation and cell-aggregate fusion, behaviors not observed by tumorigenic cells derived from breast tumors [18, 19]. In addition, a screen of 51 monoclonal antibodies against cell surface molecules identified two mAbs that blocked coalescence in the 3D Matrigel model, one against beta-1-integrin (CD29) [47], and one against CD44, the hyaluronate adhesion receptor implicated in melanoma metastasis [48–54]. We also show that the A6 peptide, which recognizes the hyaluronic acid (HA) binding site of CD44, had little or no effect on coalescence at concentrations as high as 1 mg per ml. Our results suggest that coalescence may be a general characteristic of different cancers, with minor cancer type-specific characteristics, and that the 3D model and software we have developed may not only provide new insights into the mechanisms of tumorigenesis, but also may be useful in screening for new anti-tumorigenesis drugs.

## Material and methods

### Acquisition of patient samples and cell lines

The three recurrent metastatic melanomas used in this study were isolated as follows. Melanoma 1 was isolated from an axillary lymph node of a patient originally diagnosed with melanoma in the liver. A BRAF mutation with an allele frequency of 66% was identified in genomic DNA obtained from a tumor in the liver of the same patient. Melanoma 2 was isolated through a re-excision of the left upper back of a patient with a stage IIC melanoma. Melanoma 3 was isolated from an axillary lymph node of a patient originally diagnosed with melanoma on the left lower back. Tumor specimens were initially assessed by a pathologist and the attending physicians. The tumors were clearly delineated from adjacent tissue. Samples were harvested from the flat, polyploid region of the excised specimens that were phenotypically melanoma. Fresh normal skin for isolating melanocytes was obtained from three breast reduction procedures. The adipose layer was removed by dissection. Fresh tumor biopsies and fresh normal tissues were obtained under approved protocols as follows. The melanomas from Mercy Hospital Systems, Des Moines, IA, was obtained under approval by the Western Institutional Review Board (WIRB), Protocol #20091867, with written informed patient consent to participate in a study entitled "Multi-center Collection of Biospecimens and Associated Clinical Data". Two melanomas were procured by protocols approved by the University of Iowa Institutional Review Board, IRB ID#200804792, with written informed patient consent to participate in a study entitled "Ocular Skin and Connective Tissue Proliferative Disorders, Clinical Data and Tissue Sample Collection Project", through the Melanoma and Sarcoma Tissue Bank Registry (MAST; https://www.uihealthcare.org/melanoma/) at the University of Iowa Carver College of Medicine, project number 028, Dr. Milhem. Fresh skin was obtained through the Tissue Procurement Core (TPC), a facility that manages the University of Iowa Biobank, IRB# 201103271, that is jointly supported by the Carver College of Medicine and the Holden Comprehensive Cancer Center at the University of Iowa. The HTB-66 cell line, a human malignant melanoma from a lymph node metastatic site [55, 56], and A375M, a human metastatic melanoma cell line isolated by *in vivo* selection in a mouse of metastatic cells from a population of poorly metastatic parent cells [57] were purchased from ATCC (ATCC.org) and subcultured according to ATCC-recommended methods. Adult normal human epidermal melanocytes (NHEM) were obtained from Lonza (www.lonza.com) and cultured according to the specifications provided by the company.

### Preparation of cell cultures

Melanoma samples, which were at least 1 cm in diameter were dissected into fragments approximately 3 to 4 mm in diameter and were either used directly in the 3D Matrigel model or placed in wells of a 24-well tissue culture plate for subculturing. Both preparations contained DMEM/F12 medium (ThermoFisher, Grand Island, NY), supplemented with 20 ng/ml EGF, 10 μg/ml insulin, 0.5 μg /ml hydrocortisone, 0.1 μg/ml cholera toxin (Sigma-Aldrich, St. Louis, MO), 5% horse serum (Atlanta Biologicals, Atlanta, GA), penicillin/streptomycin, fungizone (ThermoFisher) and Gentamycin (Sigma-Aldrich, St. Louis, MO). For generating secondary cultures, the medium in the wells of the multiwell plate was replaced with fresh medium after one week. Once cell growth outside the fragments was observed, the medium was changed every 3 days. Cells were harvested at ≥ 70% confluency. Cells were harvested by treating them with Dispase (Stem Cell Technologies, Vancouver, BC, Canada) to gently dissociate them from the surface of the wells and transferred into a T-75 tissue culture flask. Once cells were harvested and maintained in a tissue culture flask, they were considered secondary cultures. In the case of the melanocyte cultures we generated from skin, samples were dissected into pieces approximately 3 to 4 mm in diameter. The adipose layer was removed by dissection and the resulting samples further dissected into pieces 3 to 4 mm in diameter. These pieces, containing both dermis and epidermis were incubated in six well tissue culture plates as described for secondary cell cultures of melanoma tissue, with the same medium regime, and the cells that exited the tissues harvested at > 80% confluency. A melanocyte preparation was also purchased from the company Lonza (Walkersville, MD), and cultured in the same way as the primary cultures we generated.

### Immunofluorescent staining

The monoclonal antibody (mAb) CPTC-MAGEA4-3, against MAGEA4 and CPTC-S100A4-1, against metastasin 100 calcium binding protein A4, were developed by the NCI Clinical Proteomics Technologies for Cancer (CPTC) initiative and banked in the Developmental Studies Hybridoma Bank (DSHB) (dshb.biology.uiowa.edu), an NIH National Resource housed at the University of Iowa. The anti-fibroblast mAb CP28, against CD90/Thy-1, was obtained from Calbiochem/EMD Millipore (www.emdmillipore.com) and the mAb HMB45 against Pmel 17 from DAKO/Agilent Technologies (www.dako.com). The characteristics of these mAbs provided by the distributors of these mAbs are outlined in S1 Table. Cells from melanomas, melanoma cell lines and cultured skin melanocytes were grown overnight in Thermo Scientific Nunc Lab-Tek II chambered slides (Fisher Scientific, Pittsburgh, PA). Cultures were then rinsed 3 times with PBS, fixed with 4% paraformaldehyde for 15 minutes, briefly permeabilized in 0.2% Triton-X 100, washed in PBS, and blocked with 5% normal goat serum (NGS) in PBS. The mAbs CPTC-MAGE4-3, CPTC-S100A4-1 and CP28 were diluted to a concentration of 3 μg/mL in 5% NGS/PBS while HMB45 was diluted 1:50. All mAbs were applied for 30 mins at room temperature. Preparations were then washed, and stained for 45 mins at room temperature with goat anti-mouse Alexa 488 (www.jacksonimmuno.com) diluted 1:300. Slides were mounted in Vectashield (Fisher Scientific, Pittsburgh, PA).

### 3D Matrigel model

The 3D Matrigel model (Fig 1A) was prepared according to methods previously described in detail, using glass-windowed, modified Petri dishes for DIC microscopy [19, 31, 32]. In brief, the glass window at the bottom of a modified 65 mm Petri dish was pre-coated with a 100 μl film of Matrigel (Corning, Life Science, Corning, NJ). A 500 μl aliquot of chilled Matrigel was mixed with 250 μl of a cell suspension containing 1.25 x 10^5^ cells at 4°C or with tissue or suspension of tissue fragments. The gel was then pipetted onto the pre-coated bottom glass window. The preparation was incubated for 30 minutes at 37°C to allow gelation. The final 3D Matrigel model was approximately 1.5 mm thick. The dish was then filled with medium. Cells or fragments were distributed independently and randomly in the 3D gel. To test the effects of mAbs on coalescence in 3D, purified mAb were mixed with a 250 μl cell suspension prior to addition of 500 μl of chilled Matrigel. The final concentration of mAb in the 3D preparation was 500 μg per ml.

### Optical sectioning

Automatic and manual outlining were performed as previously described [18, 19, 31], with minor modifications. In brief, the dish containing the 3D Matrigel model was positioned on the stage of a Zeiss Axioplan 2 microscope equipped with DIC optics, a motor-driven stage and a Zeiss AxioCam MRc5 IEEE 1394 color CCD camera, all computer-synchronized (Fig 1A). The microscope was housed in an incubator, set at 37°C and 5% CO_2_. A collection of 150 DIC optical sections were obtained in a 45 second period through 1500 μm (Fig 1B), and repeated every 10 minutes. A series of DIC optical sections is presented in Fig 1C at different depths. In the previous version of DIAS, J3D-DIAS4.1[31, 32], images were converted to a DIAS format to save memory. In the latest version of the software used here, J3D-DIAS4.2, image stacks were saved in a JPEG format to generate movies with little to no compression. Thus, high resolution images, equivalent to those acquired by the camera, were obtained.

### Image processing

In the previous version of the reconstruction software, if the background had non-uniform lighting, the entire image was smoothed and the smoothed image was then subtracted from the original one. Uniform background was achieved, but contrast was sacrificed. As a result of the improved speed and performance of new generation computer processors, we implemented the “rolling ball” method [58] for background subtraction and object enhancement in the J3D-DIAS 4.2 upgrade used here, which minimizes the loss of contrast.

### Object detection (image segmentation)

Automated methods of image segmentation, including “complexity-based bitmap object detection” (C-BBOD), have been described elsewhere in detail [19, 32]. In brief, the complexity algorithm for automatically tracing a cell or aggregate in a DIC optical section assigns a 0 to 255 gray scale value to each pixel within a kernel. A kernel is a pixel matrix in an optical section image with a user-determined size (for example, 3x3, 5x5 or 7x7 pixels). The standard deviation of the average gray scale values of the pixels within the kernel is calculated and assigned to the reference pixel within the kernel. If that standard deviation is greater than or equal to the user-determined threshold, the reference pixel is considered part of the object of interest and is retained. The kernel automatically moves to the next pixel, calculations are performed and the process is repeated until every pixel has been assigned a value in the optical section. The background has more uniform gray scale values than a cell or aggregate and will therefore not be included in an object. A series of autotraced, in focus images are presented at different depths for a single reconstruction in Fig 1D. For more control of detail at the edge of the cell, like filopodia, a “detailed manual trace” option has been introduced into 3D-JDIAS4.2 that magnifies the image. As in the previous version 3D-JDIAS4.1, manual outlines, but not bitmap traces, are replaced with beta-splines [59–62].

As described previously [32, 59–65], tracings that overlap in the z-dimension are then grouped as a complexity stack in the case of bitmap tracings or as a series of outlines (beta-spline representations) for manual tracings. In the former case, the bitmap pixels become the voxels (3D pixels), while in the latter, the outlines are filled with voxels (3D pixels). In both cases, the voxel dimensions are determined by the distance between slices and the total magnification of the image. Based on these x, y, and z scale factors, a 3D voxel map (raw voxel block) is generated.

### Generating encapsulated or faceted 3D images

The stacked outlines obtained by segmentation of the DIC optical sections are faceted in 3D [59–61, 66]. The earlier versions of 3D-DIAS [59, 60, 66] used a wrapping technique which was subsequently replaced in J3D-DIAS4.1 [19] with “adaptive skeleton climbing isoform extraction” [67]. The latter technique eliminated the column artifact that occurs in wrapping, but introduces gaps between facet vertices, missing facets and backwards facets that required rendering steps to correct. The “marching cubes” algorithm [68] was therefore implemented in J3D-DIAS 4.2. In addition, to improved accuracy, we estimate that the fully implemented marching cubes algorithm renders objects 10 times faster than adaptive skeleton climbing. The faceted surface is then smoothed using a vertex smoothing algorithm [69] as previously described [19, 32]. Three rounds of vertex smoothing are generally sufficient to attenuate sharp edges in the faceted object. A rendering of a reconstructed 3D aggregate at a single time point, formed by coalescence, is viewed from four angles in Fig 1E.

### Generating 4D centroid tracks and data analysis

J3D-DIAS 4.2 was modified in order to permit the generation and visualization of 4D centroid tracks. In essence, the 2D algorithm [59–62] was ported to JAVA and generalized to 3D, a process that required storing data in a fashion that could be rapidly accessed. Once 4D tracks were generated, the newly implemented quick display feature was used to select one object in a field, and acquire data for that object in graphic as well as textual format.

### Preparation of mAbs to test for anti-coalescence

To select mAbs to use in a screen for inhibition of coalescence of tumorigenic cells, a search was first conducted using the search features on the Developmental Studies Hybridoma Bank (DSHB) website (http://dshb.biology.uiowa.edu) and the key words “cell surface”, “receptor”, “CD”, “transporter”, “exchanger, channel”, “plasmamembrane”, and “endocytosis”. In addition, antibody collections listed at the DSHB website, such as http://dshb.biology.uiowa.edu/Antibody-Collections/Cancer-antibodies, and an antigen list also available at the DSHB website, http://dshb.biology.uiowa.edu/Antigen-list, were examined for cell surface targets. mAbs were purified using Hi Trap Kits (GE Healthcare, Upsalla, Sweden). To remove salt, filter-sterilized mAbs were passed through Amicon Ultra-Cel 50K columns (EMD-Millipore, Massachusetts) and resuspended in PBS. Protein content was assessed at 280 nm using a Nanodrop 1000 spectrophotometer (ThermoScientific). Characteristics and validation of the mAbs tested in the 2D and 3D assays are presented in S2 Table.

### Screen for anti-coalescence

In a rapid screen, individual wells of a 96 well plate were coated with 50μl of Matrigel and incubated for 30 min at 37°C. Log phase HTB-66 cells were resuspended in medium at a concentration of 3x10^5^ cells per ml. The cell suspension was poured through a cell strainer with 70 μm pores (Fisher Scientific). Eighty μl of this cell suspension was mixed with 60 μl of proclin-free supernatant mAb or purified mAb at a concentration of 0.8–1.5μg/μl. Thirty μl of Matrigel were added and the suspension gently mixed. Eighty μl of this mixture was added to each pre-coated well and allowed to polymerize at 37°C for 30 minutes before addition of 100 μl of medium. The final concentrations of mAbs was 500 μg/per ml. The control was set up similarly, but beginning with 60 μl of medium lacking mAb. This preparation provided a 3D milieu, but allowed high through-put screening using 2D bright field microscopy. All mAbs were tested in duplicate wells. Images from each well were taken at three depths through five different regions every 24 hours over a period of 4 days with an Axiovert 100 inverted microscope (Zeiss), using a 10x phase objective and a Sony XCD-V50 digital camera. Growth, the formation of bridges and aggregate coalescence in mAb-treated preparations were qualitatively compared to untreated controls. mAbs that did not affect growth but blocked bridge formation and coalescence were then reanalyzed in the 3D model [18, 19] using the J3D-DIAS 4.2 reconstruction software described in preceding sections.

### A6 peptide

The peptide, obtained from Biomatik (Wilmington, DE), consisted of the eight amino acids KPSSPPEE, acetylated at the N-terminus [70]. It was dissolved in PBS to a final concentration of 100 mg per ml according to manufacturer’s instructions and diluted with media for analysis in the microwell assay to assess concentration effects and in the 3D model containing 1 mg per ml of A6 peptide.

## Results

### Demonstrating the melanoma phenotype

**Fig 2:**
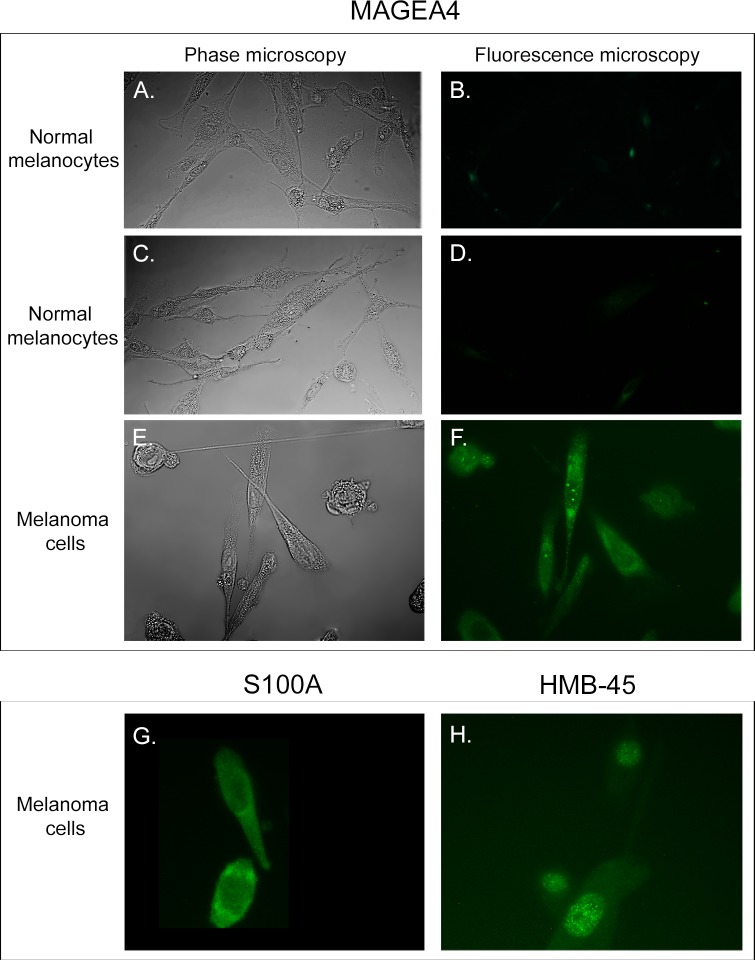
A majority of cells cultured from fresh melanomas stain for the melanoma-enhanced antigens MAGEA4-3, S100A and HMB-45. A, B and C, D. Phase contrast microscopy and fluorescence microscopy of control melanocytes cultured from the skin of two breast reduction patients reveal that these cells do not stain with the mAb CPTC-MAGE4-3 against MAGE4-3. E, F. The great majority of melanoma cells cultured from melanoma fragments stain with the anti-MAGEA4-3 mAb CPTC-MAGE4-3. G, H. The great majority of melanoma cells cultured from melanoma fragments stained with the anti-metastatin 100 calcium binding protein A4, mAb CPTC-S100 A2 and the anti-PME117, mAb HMB45, respectively.

To verify that the cells exiting fragments of fresh melanoma tumors exhibited the melanoma phenotype, we stained cells from both primary and secondary cultures for melanoma-specific antigens employing antigen-specific mAbs. The melanoma markers included MAGEA4-3 [71–73], S100A [74, 75] and HMB45 [74, 76]. Approximately 95% of cells from secondary cultures of melanoma cells derived from fresh melanoma stained for MAGEA4-3 (Fig 2E and 2F), S100A4-1 (Fig 2G) and HMB45 (Fig 2H). Control melanocytes cultured from the skin of two breast reduction patients did not stain for CPTC-MAGE4-3 (Fig 2A, 2B, 2C and 2D). Results for CPTC-S100A4-1 and HMB45 in control melanocytes were identical to those observed with MAGE4-3 (Fig 2B and 2D). Approximately 5% of cells from secondary melanoma cultures and melanocyte cultures stained with the anti-fibroblast mAb CP28, against CD90/(Thy-1) [77]. Moreover, the majority of both primary and secondary cultures contained brown pigment, indicative of melanin synthesis. These results indicated that the great majority of cells cultured from fresh tumors expressed melanoma-specific markers.

### Normal melanocytes do not coalesce

**Fig 3:**
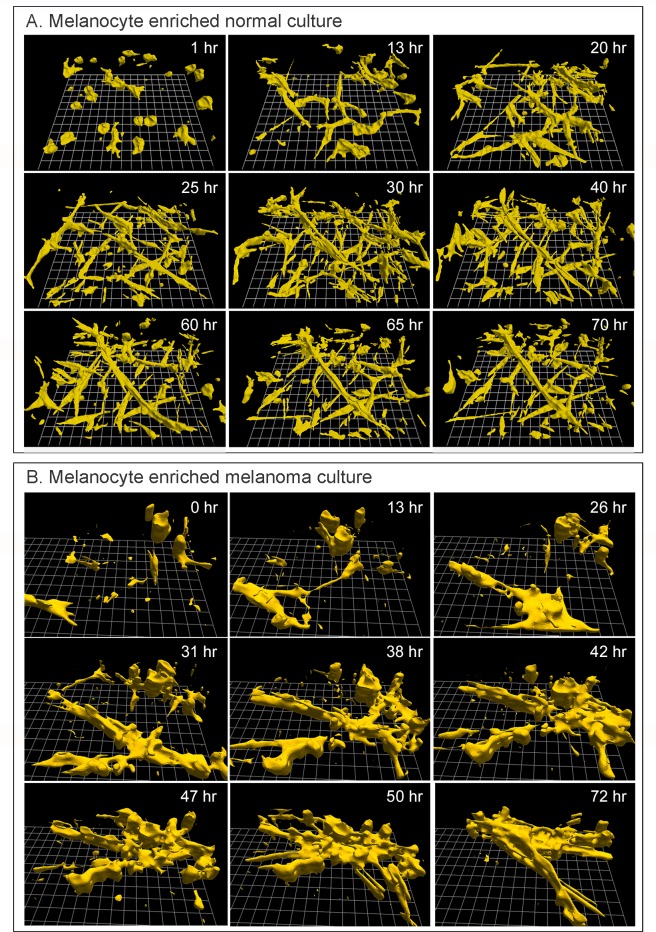
Cells in melanoma cultures, but not in melanocyte cultures undergo coalescence in the 3D Matrigel model. A. 3D reconstructions of a representative preparation containing melanocytes cultured form the skin of a breast reduction patient reveal that these cells increase in number, elongate, and form multicellular cables. They are highly motile, but do not undergo coalescence to form a single aggregate. B. 3D reconstructions of a representative preparation of melanoma cells that had exited a tumor fragment suspended in Matrigel multiply, coalesce and form a single long, flat fenestrated aggregate through coalescence. Reconstructions were started four days after gelation, which was designated zero hours in the reconstruction.

Melanocyte enriched cultures from the skin of three breast reduction patients were seeded in the 3D Matrigel model, using the same procedures used for melanoma cells. Since the cells of all three melanocyte enriched preparations behaved similarly, a time series of reconstructions of only one preparation is presented over a 75 hour period of analysis in Fig 3A. All outlining and reconstructions were performed automatically [19, 31]. The melanocytes were initially round or amorphous in shape (Fig 3, 1 hr), but became highly elongate, spindle-shaped and dendritic by 13 hours, and remained elongate through 70 hours (Fig 3A). The elongate, dendritic phenotypes were consistent with those reported for melanocytes analyzed on 2D substrates [78–80]. The melanocytes moved rapidly through the 3D Matrigel, at velocities sometimes exceeding 25 μm per hour. As the cells multiplied, they formed a dendritic network and in many instances the networks included cables of elongate cells aligned end to end (Fig 3A). The increase in cell number was apparent, when the density at 13 hours was qualitatively compared to that at 30 and 60 hours (Fig 3A). However, the melanocytes did not coalesce into single, large aggregates in the areas of analysis, even after 70 hours (Fig 3A). Commercially obtained melanocytes seeded in the Matrigel model and reconstructed in 4D exhibited the dendritic morphology and rapid motility observed in melanocytes we cultured from breast reduction tissue, and, as was the case for our melanocyte preparations, did not undergo coalescence (S1 Fig, after 150 hours).

### Cells directly exiting melanoma tumor fragments undergo coalescence

Cells exited fragments of melanomas embedded in Matrigel approximately five days after gelation. The cells spread through the Matrigel by approximately seven days, forming a number of small aggregates containing four or more cells (Fig 3B, 0 hr). The shape of the individual melanoma cells at seven days varied from round to elongate and dendritic (Fig 3B, 0 hr). Cells in the preparation continued to multiply, elongate and coalesce (Fig 3B, 0 to 72 hours). Through the 70 hours of analysis, coalescence occurred between cells, between cells and aggregates, and between aggregates (Fig 3B). At the onset of analysis, the 3D field contained 24 cells and small aggregates (Fig 3, 0 hr). By 72 hours, the field contained one large aggregate and four cells or small aggregates (72 hr, Fig 3B), an 80% reduction in independent entities resulting from coalescence of cells and aggregates (Fig 3B). It was clear that the mass of both cells and aggregates increased through cell multiplication. Because of the high rate of cellular translocation, cells continued to enter and exit the field during reconstruction. The large aggregate formed at 47 hours was highly dynamic, changing shape continually, as evidenced by visual comparisons of the aggregate at 47, 50 and 72 hours (Fig 3B). A movie of coalescence, which included 72 hours of analysis and consists of 433 reconstructions of the 3D area of analysis, is presented in S1 Movie.

### Melanoma cells subcultured from tumors

**Fig 4:**
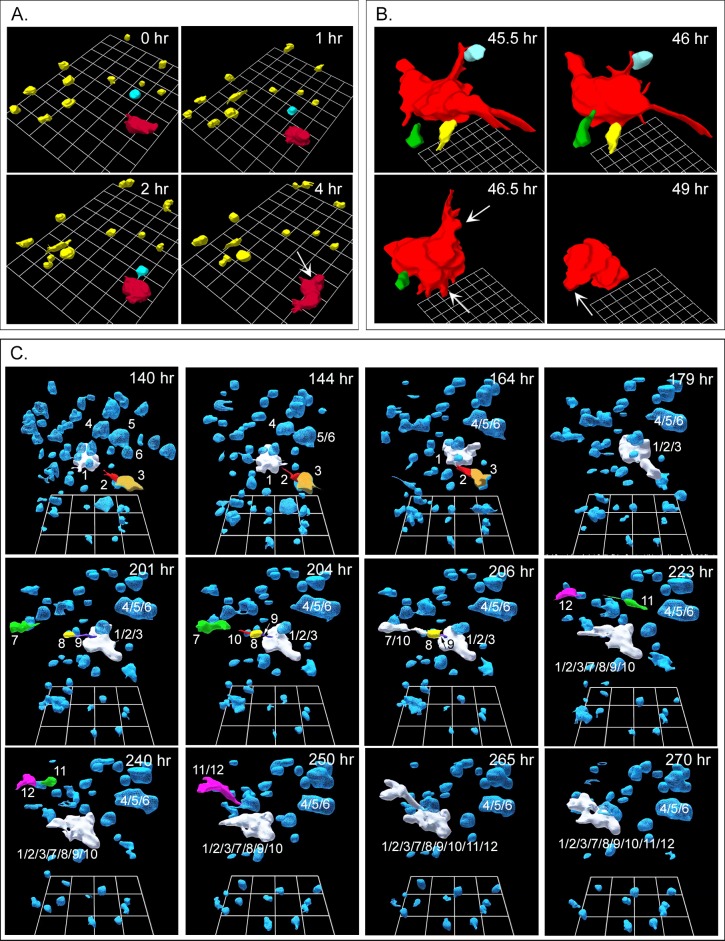
Cells subcultured from melanoma and seeded in Matrigel undergo cell-aggregate and aggregate-aggregate coalescence. The three examples in panels A, B and C were reconstructed at different times and different locations of a single preparation. A. Example of a cell (blue) translocating to a small aggregate (red) and coalescing with it, in a four hour period. B. Example of three individual cells (green, yellow, blue) coalescing with an aggregate (red), between 45.5 and 49 hours. C. Examples of cell-aggregate and aggregate-aggregate coalescence between 140 and 279 hours of reconstruction. In these panels, the original cells and small aggregates that undergo coalescence are multicolored and numbered so that they can be monitored through coalescence. Note that cells and aggregates 1, 2, 3, 7, 8, 9, 10, 11 and 12 form the aggregate labeled 1/2/3/7/8/9/10/11/12 and cells 4, 5 and 6 form 4/5/6 aggregate.

Cells subcultured from fragments of all three fresh melanomas exhibited the initial round cell phenotype of melanoma cells exiting melanoma fragments, but did not fully regain the very elongate, dendritic cell phenotype of primary cultures (Fig 4A, 4B and 4C). Nonetheless, they still underwent coalescence. As previously noted, these cells stained for melanoma-specific antigens (Fig 2). The dynamics of coalescence of subcultured melanoma cells exhibited specific differences from the dynamics of cell lines derived from breast tumors [18, 19]. When seeded into the Matrigel model, breast tumor-derived tumorigenic cells underwent cell multiplication for approximately 100 hours, exhibiting little translocation and, through cell multiplication, formed clonal islands, or clonal aggregates [18, 19]. It was only at the end of this precoalescence period, lasting approximately 100 hours, that specialized cells (facilitators, probes) appeared and began to mediate coalescence between aggregates [18, 19]. In marked contrast, subcultured melanoma cells underwent coalescence immediately after Matrigel gelation and in parallel with cell multiplication (Fig 4A, 4B and 4C). In other words, there was no initial precoalescence period for melanoma cell cultures during which only clonal cell aggregates formed in the absence of coalescence through cell multiplication. The presence of small aggregates immediately after Matrigel gelation also suggested that in melanoma cell preparations, coalescence had begun during gelation. Moreover, in contrast to cultures of breast-derived tumorigenic cell lines, coalescence of subcultured melanoma cells initially occurred between single cells as well as between single cells and the small aggregates formed during gelation (Fig 4A). In the three examples of coalescence in Fig 4, the times of reconstructions represent the period after gelation. In the representative sequence of 3D reconstructions over an initial four hour period beginning at zero hours in Fig 4A, a single cell, color-coded blue, migrated over a distance three times its diameter, in a directed fashion (see arrow in Fig 4A, 4 hr) towards a small aggregate color-coded red, and coalesced with it by four hours. Coalescence of a single cell to another cell or aggregate is evident in 3D reconstructions between 45.5 and 49 hours in a different preparation of the same subcultured melanoma cells (Fig 4B). In panels A and B in Fig 4, cell regions were selected to demonstrate cell-aggregate coalescence. In Fig 4C, all cells and aggregates in a region of analysis of subcultured melanoma cells were reconstructed from 140 hours post gelation in order to demonstrate the extent of coalescence. Those initial cells and aggregates that underwent coalescence were numbered in order to track the process. The original aggregate 1, which served as a major focal point for coalescence, was color-coded white. As single cells (color-coded other than white) and small aggregates (color-coded other than white) coalesced with aggregate 1, they lost their original color code (Fig 4C) and assumed the white color of aggregate 1. The generation of aggregate 1/2/3/7/8/9/10/11/12 can thus be tracked through the period of analysis, between 140 and 270 hours (Fig 4C). The generation of the small aggregate 4/5/6 can also be tracked between 140 and 164 hours (Fig 4C). Examples of cell-cell (11 and 12), cell-aggregate (8 and 1/2/3) and aggregate-aggregate (11/12 and 1/2/3/7/8/9/10) coalescence were verified by rotating the reconstructed sequence.

### High resolution analysis of single cell-aggregate coalescence

**Fig 5:**
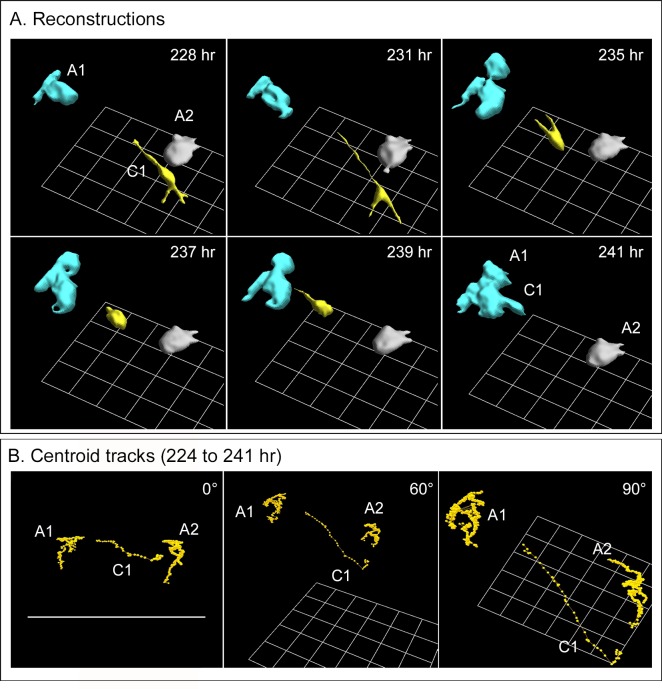
Tracking of a single cell to an aggregate and subsequent coalescence. The preparation was that of a secondary culture of melanoma cells derived from a fresh melanoma fragment. A. 3D reconstructions of a single cell (yellow, C1) in the neighborhood of two aggregates (blue, A1; grey A2) that extends three processes, translocates in the direction of the extension directed towards the blue aggregate, A1, and coalesces with it. B. Centroid (center of mass) tracks of the cell C1 and the aggregates A1 and A2, all reconstructed in panel A, between 224 and 241 hours, reveal directed translocating of the cell towards aggregate A1 prior t o coalescence.

To obtain high resolution reconstructions of coalescence between a single melanoma cell and an aggregate, a cell, color-coded yellow, was manually traced and two neighboring small aggregates (blue and grey) autotraced (Fig 5A and 5B). At 228 hours from the time of gelation, the individual yellow cell had extended three protrusions, two away from the two aggregates and one towards the blue aggregate. No protrusions formed towards the grey aggregate. The protrusion towards the blue aggregate ended in a thin filopodium that could not be accurately reconstructed at its very end because of its small diameter, estimated to be less than 0.1 μm (Fig 5). The cell then migrated along the path of the filopodium towards the blue aggregate, bypassing the grey aggregate. By 241 hours, the cell had attached to and coalesced with the blue aggregate, and its color-code changed to blue (Fig 5A).

Because the J3D-DIAS4.2 program converts cells and aggregates into mathematical models, 3D motility parameters are readily computed. In Fig 5B, tracks of the cell centroid (the center of mass) [59, 60] and those of the two aggregates are plotted in 3D at 15 minute intervals between 224 and 241 hours for the yellow cell (C1) and the blue and grey aggregates (A1, A2), and presented at three angles, which can be assessed by the position of the bottom grid. The centroids of the two aggregates clustered (Fig 5B), suggesting random movement of the centroids due presumably to shape changes effected by the motile behavior of cells within the aggregates. In marked contrast, the centroid of the cell (C1) clustered between 224 and 227 hours, then between 227.5 and 240 hours translocated in a directed fashion towards aggregate A1 (blue) (Fig 5B). The centroid then clustered between 240.5 and 241 hours at the edge of the targeted aggregate, during coalescence (Fig 5B). This scenario of facilitation was observed for many additional cells coalescing with aggregates in preparations from all three melanomas, which were similarly analyzed in 3D.

### Single cell facilitation of aggregate-aggregate coalescence

**Fig 6:**
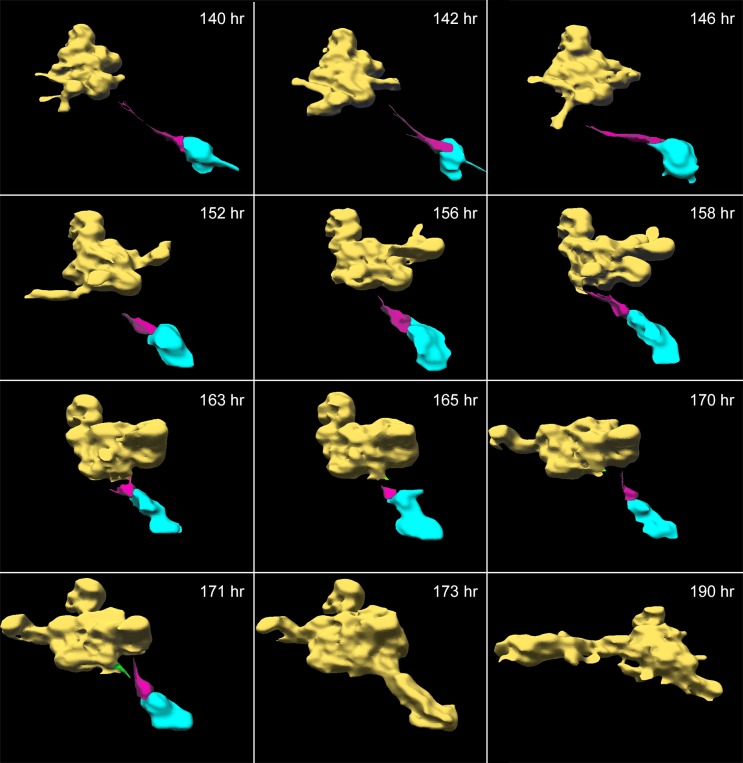
Single cell facilitation of coalescence between two aggregates. The preparation was that of a secondary culture of melanoma cells derived from a melanoma biopsy. The single facilitator cell (magenta), attached to the small aggregate (blue), extended a tapering projection ending at the large aggregate (yellow). The large aggregate extended a green projection at 171 hours which in the direction of the cell, then attached to it. The projection of the magenta facilitator cell attached to another cell on the surface of the large yellow aggregate and contracted as a multicellular bridge formed (not shown), resulting in coalescence of the two aggregates. The small blue aggregate was incorporated into the large yellow aggregate by 173 hours, and was then color-coded yellow.

In 3D Matrigel models seeded with tumorigenic cell lines derived from breast tumors, aggregate coalescence was demonstrated to be mediated by single specialized “facilitator cells” and “probes” [19]. Attachment of facilitators to probes or between probes of different aggregates initiates additional cells to extend along the attachment to form a multicellular cable that then contracts, always pulling the smaller aggregate into the larger one [19]. A similar mechanism was observed in the mediation of aggregate-aggregate coalescence in 3D melanoma cell preparations. A representative example is presented in the reconstructions in Fig 6. In this example, a single cell, color-coded magenta, mediated coalescence between a large aggregate, color-coded yellow, and a small aggregate, color-coded blue. The aggregates were autotraced and the cell manually traced. The facilitator cell then sent a single tapering projection in the direction of the large yellow aggregate (Fig 6, 140 hr). Between 140 and 171 hours, the projection appeared to probe the surface of the large, yellow aggregate. At 171 hours, a probe cell at the surface of the large aggregate, color-coded green, sent out a projection that contacted the magenta-colored cell (Fig 6, 171 hours). Additional cells from the large aggregate rapidly moved into the junction to form a cable, which then contracted (173 hours), then drew the small blue aggregate into the larger yellow aggregate (Fig 6, 190 hr). It should be noted that in this example the aggregate and facilitator began moving towards the large aggregate before cable formation, following the path of the projection, which decreased in length, as if by contracting, it was drawing the smaller to the larger aggregate.

**Fig 7:**
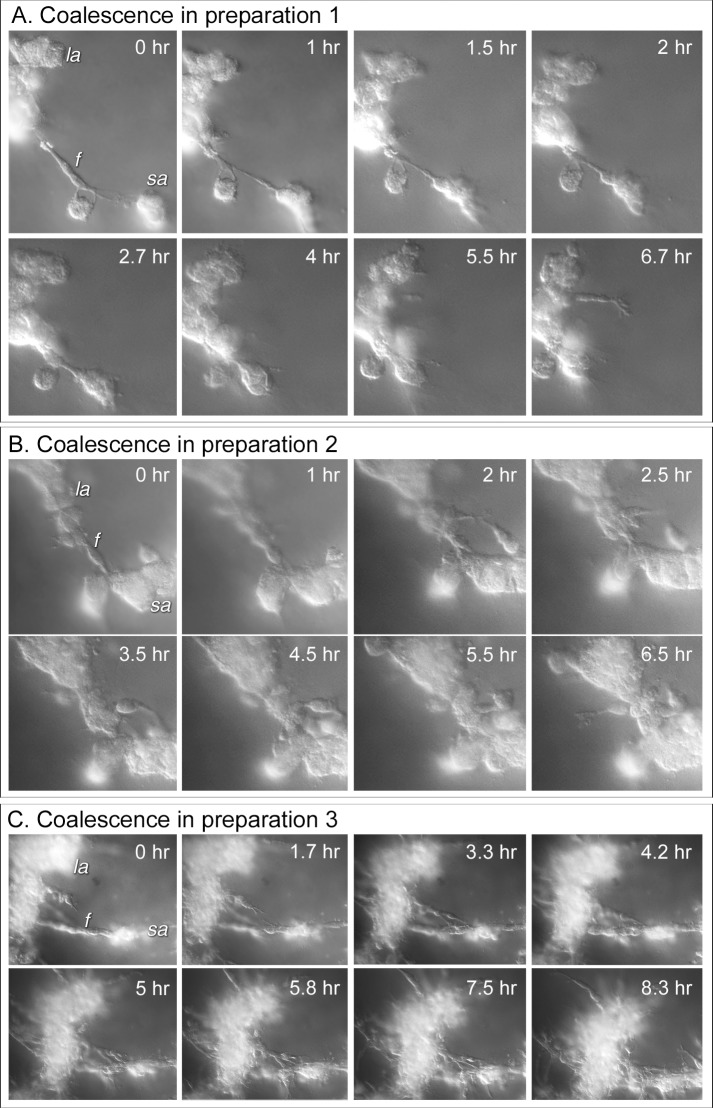
Differential interference (DIC) microscopy images over time of representative bridges formed by facilitator cells to move small aggregates into large aggregates in the process of coalescence. The time series of optical sections in each of the three examples was taken at one depth over time for comparison. A. In each example, a single or multiple faciltator (f) cells connect a small (sa) to a large aggregate (la), and through contraction of multicellular cable moves the small into the large aggregate, mediating coalescence. B. A single facilitator (f) cell connects a small (sa) and large (la) aggregate. Additional cells extend out of the small aggregate, join the facilitator cell, forming a cable that contracts, moving the small to large aggregate. C. Multiple facilitator cells form and cable end to end. Additional cells join the cable, then contract, bringing the small into the large aggregate. The large aggregates are not in view in panels B and C.

Three additional examples of the mediation of aggregate-aggregate coalescence by facilitator cells are presented in time sequences of DIC optical sections viewed at a single depth in Fig 7. In the first example in Fig 7A, a single elongate bipolar facilitator cell (f) attached to a large aggregate (la) and a small aggregate (sa) then contracted, drawing the smaller into the larger. In the second example in Fig 7B, a single elongate bipolar facilitator cell (f), positioned between a small aggregate (sa) and a large aggregate (la), recruited cells from both the small and large aggregate, forming a cell cable that contracted, drawing the small aggregate into the larger one, which for the most part is to the right of the image, out of the field of view. In the third example in Fig 7C, a linear cable of multiple facilitator cells aligned end to end, formed between a small aggregate (sa) and a large aggregate (la). The latter is to the right of the image and, for the most part, out of view. Cells from the small aggregate extended out and along the cable (Fig 7C, 0 to 7.5 hr), then joined with the cable, generating a thicker cable between the aggregate. The cable then contracted, pulling the small aggregate into the large one (Fig 7C, 7.5 to 8.3 hr).

### Coalescence of melanoma cell lines

**Fig 8:**
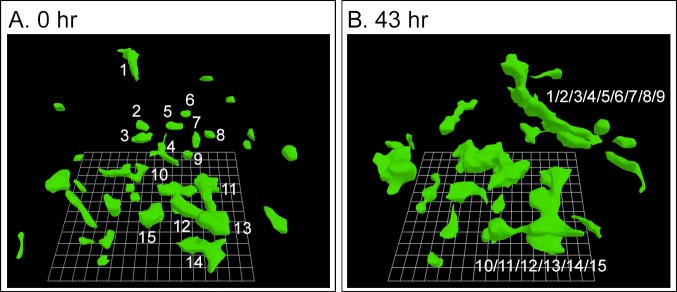
Coalescence of cells of the established melanoma cell line HTB-66 begins immediately after Matrigel coalescence and is rapid. Only the cells that coalesced into large aggregates in the 43 hour period of reconstruction are numbered.

Since the original studies of coalescence employed tumorigenic cell lines derived from breast tumors and since any screens developed for anti-coalescence drugs, required an established immortalized melanoma cell line, we tested coalescence in two melanoma cell lines, HTB-66 [55, 56] and A375M [81, 82], for use in a screen for mAbs that blocked coalescence. The cell line HTB-66 underwent rapid coalescence (Fig 8). HTB-66 cells were round to elongate in the 3D Matrigel model (Fig 8). Coalescence began immediately after Matrigel gelation, just as it did for secondary cultures derived from fresh melanoma preparations (Fig 3). In the 3D preparation in Fig 8, the number of individual cell cables decreased from 31 to 13 through coalescence in a 43 hour period, a decrease of 60%. We also found that the cell line A375M underwent coalescence, but the rate was less than half that of HTB-66 cells, with far fewer identifiable facilitator cells. We therefore chose HTB-66 cells for the screen.

### Final aggregate shape in the 3D Matrigel model

The tumorigenic cell lines derived from breast tumors eventually formed large sessile spheroids with highly ordered architecture [19]. In marked contrast, the final aggregates formed through coalescence of melanoma cells exiting fragments of melanomas, secondary cultures of cells exiting tumors and HTB-66 cells remained fenestrated, amorphous, and highly active. They changed shape continuously, translocated, and distorted the Matrigel. They usually exited the field of analysis as a result of their highly mobile behavior and gel distortion. In addition, they assumed an elongate, relatively flat morphology, as is evident in the preparations in Fig 3B at 72 hours and Fig 6 at 190 hours.

### Inhibition of coalescence by anti-ß-integrin and anti-CD44 mAbs

We previously demonstrated, using tumorigenic cell lines derived from breast cancers, that the mAb AIIB2, against ß-1integrin (CD29), did not inhibit cell multiplication and the formation of clonal aggregates resulting from cell multiplication in the 3D Matrigel model using the tumorigenic mammary tumor-derived line MDA-MB-435-Br1 [19]. However, AIIB2 did selectively block the appearance of facilitator and probe cells, and inhibited coalescence of the clonal aggregates that formed.

Using a newly developed rapid protocol, not involving 4D reconstruction with J3D-DIAS 4.2 software, to assess coalescence, we screened 51 well characterized mAbs (S2 Table) for their capacity to inhibit coalescence of the melanoma cell line HTB-66. In the protocol, cells were suspended in 3D Matrigel in the wells of a 96 well plate, and each well preparation treated with a different mAb in replicate. The mAb concentrations in the well were 0.5 mg per ml. Controls were untreated. Micrographs were collected at three depths through the Matrigel at five locations in each of the two preparations for each mAb beginning at zero hours and repeated every 24 hours for four days. In control cultures, the concentration of cells and small aggregates at day one was 100 ± 25 (N = 5) per field. After four days, the concentration of individual entities (cells, aggregates) decreased to 5 ± 1 (N = 5) per field. Concentrate with more than a 90% decrease in the concentration of cells and small aggregates, the size of the aggregate per field increased dramatically, the result at least in part of the coalescence process. The average number of bridges between aggregates at four days in control preparations was 5 ± 1 (N = 5) per field. Cells in the amorphous aggregates did not exit the coalesced aggregates, once they entered, a characteristic of the aggregates formed by the coalescence of tumorigenic breast tumor-derived cells. Forty nine of the 51 mAbs did not affect coalescence in 3D Matrigel preparations. Qualitatively, four day cultures were undistinguishable from those of untreated control cultures. All exhibited an increase in culture density, a decrease in the concentration of entities (cells and aggregates), formation of bridges between aggregates and formation of a few large aggregates that dominated the field by four days. A quantitative analysis of the preparations of 10 random mAb-treated preparations having no apparent effect revealed an average decrease in entities of 94% and frequency of 6.2 ± 1.09 bridges at four days, measures very close to that of untreated controls. Two of the 51 mAbs, however, did inhibit coalescence in the screen, and did so completely, H4C4 against CD44, the hyaluronic acid receptor, and AIIB2, against beta-1-integrin (CD29), the mAb previously demonstrated to block coalescence of tumorigenic breast tumor-derived cells [18, 19]. Neither mAb appeared to have an effect on cell multiplication, as demonstrated in S2 Fig. Cell density increased in both AIIB2- and CD44-treated preparations in the absence of coalescence as demonstrated in DIC and phase contrast images (S2 and S3 Figs). In the absence of 0.5 mg per ml of the mAb AIIB2 in the 3D Matrigel model using HT66 cells, facilitator cells did not form, cables between aggregates did not develop and coalescence did not occur (S3 Fig).

**Fig 9:**
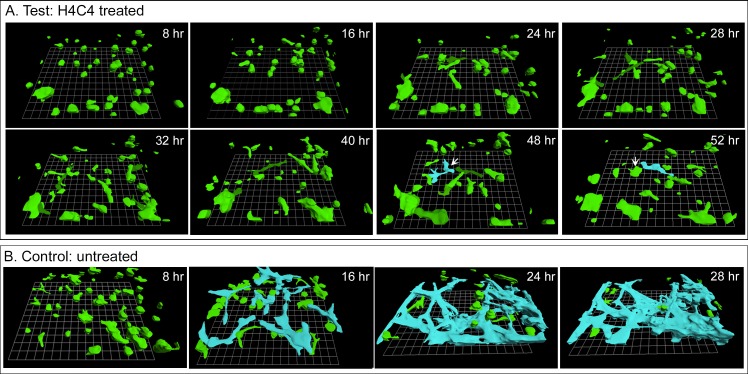
Treatment with H4C4 also blocked coalescence of melanoma cells exiting fresh melanoma tissue in the 3D Matrigel model. A. Only one potential coalescence event was observed in the treated preparation (white arrow, 48 hr), but the bridge that formed did not fully did not pull the smaller into the larger aggregate and the cells separated at 52 hr (white arrow, 52 hr). B. Coalescence in the absence of mAb. Coalesced cells are color-coded blue in panel B.

In addition to testing the effect of H4C4 on coalescence in the HTB-66 cell line, we also tested the effect of this mAb on the coalescence of melanoma cells subcultured from tumors in the 3D Matrigel model with J3D-DIAS 4.2 reconstruction software. In the presence of 0.5 mg per ml of H4C4 in the 3D model, single melanoma cells, and the small aggregates that had formed during Matrigel gelation (Fig 9A, 8 hours) remained independent through at least 52 hours of incubation. At 48 hours, some melanoma cells had made contact and appeared to coalesce (white arrow, blue color-coding) but this aggregate disintegrated by 52 hours as cells exited it (white arrow, 52 hours), indicating that, in fact coalescence had not occurred. Comparison of the eight and 52 hour reconstructions revealed major increases in the volume of the noncoalescing growth islands. In contrast, the majority of small melanoma aggregates and cells in untreated 3D models had coalesced by 28 hours into one large, stable aggregate that is color-coded blue in the field in Fig 9B. H4C4 at 0.5 mg per ml had no effect on normal melanocytes from breast reduction patients. In the 3D model in the presence of H4C4, normal melanocytes elongated, formed end-to-end contacts and established a dendritic network, exhibiting a behavior indistinguishable from normal, untreated melanocytes (Fig 3A).

### The A6 peptide did not inhibit coalescence

**Fig 10:**
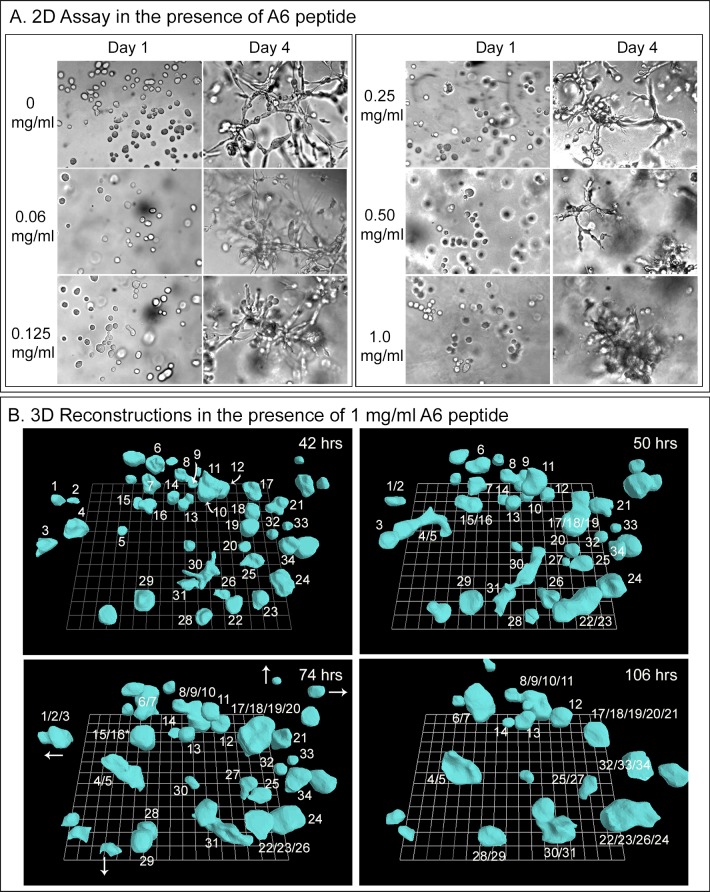
The peptide A6 had no effect on the coalescence of HTB-66 cells in the 3D Matrigel model. A. DIC images of the absence of an effect on coalescence in the 3D Matrigel model in the wells of multiwell tissue culture dishes, by peptide concentrations ranging from 0 to 1 mg per ml after four days. B. 3D reconstructions of coalescence in the presence of 1 mg per ml between 42 and 106 hours of incubation in the 3D Matrigel model reveals continued coalescence. Curved white arrows in 42 hour panel point to aggregates that are partially obscured by another aggregate. Straight white arrows at 74 hours indicate the direction of travel of cells or aggregates that moved out of the field of view.

The peptide A6 consists of 8 amino acids with homology to a sequence in the hyaluronic binding site of CD44 [83–85]. It has been shown to inhibit the migration of CD44-expressing cells of ovarian cancer cell lines as well as metastasis by B16-F10 cells, a murine melanoma cell line [84]. The A6 peptide was first tested in two independent experiments in the 2D Matrigel screening assay, employing cells of the HTB-66 melanoma cell line. Concentrations of A6 peptide ranging from 20 ng to 1 mg per ml, added to the cells prior to mixing with the Matrigel, had no apparent effect on culture growth or coalescence (Fig 10A). In the 3D Matrigel preparation of HTB-66 cells treated with 1 mg per ml of peptide added prior to addition of Matrigel, and reconstructed over a 62 hour period, there was no observable effect on coalescence (Fig 10B).

## Discussion

We previously demonstrated that cells of tumorigenic cell lines derived from breast tumors, when seeded in a 3D Matrigel model, initially undergo cell multiplication without translocating through the gel, forming relatively static clonal aggregates by approximately 100 hours [18, 19]. After this growth phase, these aggregates undergo coalescence, mediated by specialized cells, which leads to the formation of large, highly structured spheroids [18, 19]. Because the capacity to undergo coalescence occurs exclusively in tumorigenic lines and not in non-tumorigenic lines, because adding 10% of cells from a tumorigenic line to 90% of cells from a non-tumorigenic line causes the latter to undergo coalescence [18], and because specialized cells appear in the clonal aggregates after 100 hours, which mediate coalescence, we considered the possibility that these behaviors were related to tumorigenesis *in vivo* [18, 19]. Here, because of clinically observed histological data on melanomas, we have tested whether tumor-derived melanoma cells exhibit coalescence in the 3D Matrigel model. We analyzed cells derived directly from three fresh melanomas, melanoma cells subcultured from the three melanomas, as well as an established tumorigenic cell line derived from a melanoma. It is demonstrated that all of these melanoma cell preparations, but not control melanocytes derived from normal skin or commercially available melanocytes, undergo coalescence and contain specialized cells that facilitate the process in the 3D Matrigel model. However, there are also fundamental differences between melanoma-derived and breast-derived cells, some of which may relate to the different cell type origins of the alternative neoplastic cells.

### Cell preparations

The melanocyte cell preparations we generated consisted predominately of melanocytes, based on their cell morphology in Matrigel and the formation of dendritic networks, which were highly similar to the cell morphologies and dendritic networks of melanocytes cultured on a 2D substratum [78, 79]. By cytostaining, however, roughly five percent of the preparations were fibroblasts. Contamination by keratinocytes was not ruled out, but would have been minimal, given that cultured keratinocytes exhibit a uniformly spread, wide cell body, a broad, ruffling lamellipod [86] and relatively slow rates of translocation [87, 88]. Moreover, the melanocytes we isolated behaved similarly to those in melanocyte cultures purchased from Lonza, a company expert in culturing highly enriched primary human cell types (http://www.lonza.com), behaved identically to the melanocyte preparations we generated and as was the case, for our melanocyte cultures, did not undergo coalescence.

The melanoma cell preparation we generated were from the flat polypoid region of the three excised tumors of different patients and did not include normal, adjacent tissue. The great majority of melanoma cells (~95%), both in primary and secondary cultures, stained for three melanoma markers but not for a fibroblast-specific marker. Approximately five percent of cells in both primary and secondary culture did stain for the fibroblast-specific marker, indicating a low level of contaminate. The great majority of cultured cells were pigmented brown, indicating they contained melanoma. In the 3D Matrigel preparations, the cells assumed spindle and dendritic shapes and coalesced. The fresh melanoma cell preparations assumed the same shapes and formed the same large aggregates as the melanoma cell line [78, 79]. These combined results indicate that the melanocytes used as controls and the great majority of cells cultured from skin were melanocytes and from melanomas, melanoma cells.

### Absence of a sessile growth phase preceding coalescence

During embryogenesis, melanocytes differentiate from neural crest cells [89] and, consistent with the migratory phenotype of these precursor cells [90, 91], retain the capacity to crawl in the adult [92]. Likewise, melanoma cells have been shown to be highly motile, especially in wound healing assays [93–95], although this assay measures collective cell migration, a complex process that includes, but does not exclusively depend on, the motility of leader cells [96–99]. We found that melanoma cells exiting fresh melanoma tumor fragments, cells from secondary cultures of melanomas and cells of the melanoma line HTB-66, all moved in the 3D Matrigel model without a 100 hour sessile cell multiplication period, which was characteristic of breast tumor-derived cell lines [18, 19]. The sessile, clonal aggregates formed by breast tumor-derived cell lines moved after a 100 hour multiplication period, but the movement was mediated by specialized facilitator and probe cells. In marked contrast, melanoma cells were motile immediately when entering Matrigel from tumor fragments, and immediately after the one hour period of Matrigel gelation, in the case of secondary cultures of melanoma cells and HTB-66 cells.

### Immediate coalescence

In Matrigel preparations of breast tumor-derived cells, coalescence begins after 100 and 170 hours for the cell lines MDA-MB-435-Br1 [19] and MoVi-10ˈ [18]. Facilitator cells and probes appear at the surface of the aggregates at this time. Facilitator cells represent only a minority of breast tumor-derived cell line preparations. Probes, on the other hand, are numerous and appear around the aggregate surfaces of breast tumor-derived cell line preparations, only exiting the aggregate to form inter-aggregate cables. In marked contrast, coalescence began immediately in 3D Matrigel models of cultured melanoma cells from all three tumors and the cell line HTB-66. Coalescence between aggregates was mediated by individual cells that behaved in a manner very similar to facilitator cells and probes in breast tumor-derived cell lines in the 3D Matrigel model. These cells mediated the formation of inter-aggregate cables that contracted, drawing small aggregates into larger ones, as was the case for breast tumor-derived cell lines [18, 19].

### Single cell-single cell and single cell-aggregate coalescence

Coalescence of breast tumor-derived cell lines in the 3D Matrigel model occurred only between aggregates, [18, 19]. Coalescence between single cells and between single cells and aggregates was not observed, possibly because the majority of single cells had multiplied into clonal aggregates during the latency period preceding the appearance of facilitator cells and probes, and the onset of coalescence. In marked contrast, coalescence between single cells, and between single cells and aggregates, occurred from the time of Matrigel gelation and in all melanoma cell preparations.

### Specialized facilitator cells and aggregate coalescence

For cell lines derived from breast tumors, facilitator cells individually exited aggregates and aggressively initiated cable formation, but the number of facilitator cells was minimal [19]. In melanoma cell preparations, it appeared as if a greater proportion of cells, if not all, could act as facilitators and initiate coalescence. However, there is a caveat. Coalescence in breast tumor-derived cell line preparations can also be mediated by probe cells at the aggregate surfaces in the absence of a facilitator cell. A majority of cells at the aggregate surface extend protrusions that mediate cable formation, but the probe cells do not exit the aggregate until a cable is initiated. The abundance of probe cells at the surface of an aggregate suggests that after the latency periods of breast tumor-derived cell lines, probe cells may be as abundant as facilitator cells are in early melanoma preparations. It is just the timing that is different. Moreover, addition of 10% of tumorigenic cells derived from breast tumors to a non-tumorigenic 3D Matrigel model results in maximum coalescence of the latter [18], again supporting the idea that all tumorigenic cells derived from breast cancer may function as facilitators. Regardless of these considerations, it is clear from the results presented here that while specialized cells mediating coalescence appear in breast tumor-derived preparations only after a long latency period, they are present at time zero in melanoma cell preparations.

### Effects of anti-beta-1integrin and anti-CD44 mAbs

Weaver et al. [100] first showed that the anti-beta-1 integrin (CD29) mAb AIIB2 disrupted aggregate architecture of human breast cells in a 3D EHS (Engelbrete-Holm-Swarm) matrix, similar to Matrigel. We then demonstrated that the same mAb inhibited the coalescence of the tumorigenic breast tumor-derived cell line MDA-MB-435-Br1 [101, 102] in our 3D Matrigel model. It did not inhibit growth of clonal aggregates, but blocked appearance of facilitator and probe cells, so cables, which mediate coalescence, did not form [19]. We show here that the mAb AIIB2 also inhibits coalescence between cells of the HTB-66 melanoma line and aggregates in the 3D Matrigel model without affecting cell multiplication, indicating that beta-1 integrin may play a general role in the coalescence process of tumorigenic cells from different cancers. Beta-1 integrin forms heterodimers with other integrins and has been implicated in a variety of functions [103, 104], most notably as receptors for extracellular matrix molecules, including collagen, a component of Matrigel [105–108] (www.corning.com).

We also found in a 2D screen of 51 mAbs, including AIIB2, against cell surface-associated molecules using the melanoma cell line HTB-66 as well as fresh melanoma, that the anti-CD44 mAb H4C4 blocks coalescence, and this observation was repeated in 4D reconstructions in the 3D Matrigel model. In the screen, the mAbs were used at a concentration of approximately 0.5 mg per ml, which is in the range of mAb concentrations based on blood volume used in some studies of the effects of mAbs on tumorigenesis in the mouse model [109]. Guo et al. (1994) [110] previously showed that the anti-CD44 mAb, GKW.A2, inhibited melanoma metastasis in SCID mice. However, Matrigel, which is made up primarily of laminin, collagen and entactin, has only trace amounts of hyaluronic acid [106] (www.corning.com). Therefore, blocking coalescence with the anti-CD44 mAb H4C4 may not be due to an interaction with hyaluronic acid, unless the interaction is autocrine in nature–i.e., the melanoma cells secrete the hyaluronic acid [111, 112] with which they then interact. More likely, the blocking effect may be due to another CD44 function not involving hyaluronic acid. Indeed, CD44 has been shown to play a role in a diverse variety of processes related to human disease [113]. The complexity of function is manifested by the large number of isoforms of CD44, which contains 20 exons [114, 115]. We are presently determining which isoform(s) expressed on fresh melanoma cells are recognized by H4C4. H4C4 has been shown to bind to an epitope containing the hyaluronic acid binding site [116].

### The absence of an A6 effect

A6 (acetyl-KPSSPPEE) is a synthetic peptide that has been shown to affect CD44-dependent adhesion, migration and metastasis [84]. It has been shown to bind to the hyaluronic acid binding site of CD44. However, it was found to have little effect in the progression of carcinomas of the ovary, fallopian tube or primary peritoneum [117]. We found that A6 had no effect on the growth, motility or coalescence of HTB-66, although coalescence was inhibited by the anti-CD44 mAb H4C4 in these cells.

### Coalescence as a general trait of tumorigenic cells

We initially described coalescence in the 3D Matrigel model based upon our detailed analysis of breast tumor-derived tumorigenic cell lines [18, 19]. In this model, non-tumorigenic cell lines and fresh breast tissue cell culture did not undergo coalescence [18, 19]. In the process of coalescence in these preparations, single cells in Matrigel grew into clonal islands during a precoalescence period. At the end of this period, specialized cells formed multicellular cellular cables between aggregates that then contracted in each case moving the small to large aggregate, which then fused to form a bigger aggregate. This process continued until most of the clonal aggregates in a field had entered one large aggregate that then differentiated into a highly structured, hollow spheroid [18]. In melanoma coalescence, there was no precoalescence period, and cells aggregated with cells as well as aggregates, and specialized cells formed bridges between aggregates that contracted, pulling small aggregates into larger ones, culminating in a large flat, fenestrated aggregate that dominated the 3D field. Therefore, although both breast-derived cells undergo coalescence in the Matrigel model, and normal cells do not in both cases, there are obviously cancer-specific differences in the processes. However, there is commonality not only in the general process of coalescence, but also in the specificity of the mAbs that inhibit the process.

## Supporting information

S1 Fig J3D-DIAS 4.2 reconstructions of pure cultures of adult normal human epidermal melanocytes (NHEM) seeded in 3D Matrigel over a 150 hour time period. These cells exhibit the same dendritic morphology and rapid motility as melanocytes from normal skin tissue and, like melanocytes from normal skin, do not coalesce.
(TIF)

S2 Fig The H4C4 and AIIB2 antibodies inhibit coalescence in the HTB-66 melanoma cell line, but cell division proceeds. A. A representative time sequence of DIC images of a single cell taken at one depth in a 3D Matrigel culture in the presence of the H4C4 mAb reveals cell growth, division and cytokinesis by 20 hours. Cell division occurred in a majority of cells in these preparations. B. Cell division is visible in a clonal island of HTB-66 cells taken at one depth in a 3D Matrigel culture in the presence of the H4C4 mAb. Dotted black lines in the second row highlight the cleavage furrows at the time points given in the upper row. C. J3D-DIAS4.2 calculations of the volume increase over time of the clonal island in B support the conclusion that cell division is continuing in the presence of the H4C4 mAb. D. DIC images of a single cell taken at one depth in a 3D Matrigel culture of HTB-66 cells in the presence of the AIIB2 mAb reveal cell division. Scale bars are in the lower left of the first panel in each DIC series.
(TIF)

S3 Fig The mAb AIIB2 inhibits coalescence in the HTB-66 melanoma cell line. A. Brightfield images of untreated and AIIB2 treated HTB-66 cells in the 2D screen show that coalescence is inhibited through Day 3. B. J3D-DIAS4.2 reconstructions of HTB-66 cells in the 3D Matrigel culture over a 48 hour period in the presence of the mAb AIIB2 reveal that coalescence is inhibited.
(TIF)

S1 Movie J3D-DIAS 4.2 4D reconstruction of cells exiting a melanoma tumor fragment embedded in a 3D Matrigel matrix reveals rapid coalescence into a single large aggregate. (MOV)

S1 Table mAbs used to stain cells for melanoma phenotype. (PDF)

S2 Table mAbs from DSHB used to screen for inhibition of coalescence. (PDF)

## References

[1] Hanahan D, Weinberg RA. The hallmarks of cancer. Cell. 2000;100(1):57–70. 10647931 10.1016/s0092-8674(00)81683-9

[2] Hanahan D, Weinberg RA. Hallmarks of cancer: the next generation. Cell. 2011;144(5):646–74. 10.1016/j.cell.2011.02.013 21376230

[3] Pardee AB. G1 events and regulation of cell proliferation. Science. 1989;246(4930):603–8. 2683075 10.1126/science.2683075

[4] Perona R. Cell signalling: growth factors and tyrosine kinase receptors. Clin Transl Oncol. 2006;8(2):77–82. 16632420 10.1007/s12094-006-0162-1

[5] Rodeck U. Growth factor independence and growth regulatory pathways in human melanoma development. Cancer Metastasis Rev. 1993;12(3–4):219–26. 8281609 10.1007/BF00665954

[6] Waterfield MD, Scrace GT, Whittle N, Stroobant P, Johnsson A, Wasteson A, et al Platelet-derived growth factor is structurally related to the putative transforming protein p28sis of simian sarcoma virus. Nature. 1983;304(5921):35–9. 6306471 10.1038/304035a0

[7] Fisher DE. Apoptosis in cancer therapy: crossing the threshold. Cell. 1994;78(4):539–42. 8069905 10.1016/0092-8674(94)90518-5

[8] Lowe SW, Bodis S, Bardeesy N, McClatchey A, Remington L, Ruley HE, et al Apoptosis and the prognostic significance of p53 mutation. Cold Spring Harb Symp Quant Biol. 1994;59:419–26. 7587096 10.1101/sqb.1994.059.01.047

[9] Wyllie AH, Kerr JF, Currie AR. Cell death: the significance of apoptosis. Int Rev Cytol. 1980;68:251–306. 7014501 10.1016/s0074-7696(08)62312-8

[10] Doganer BA, Yan LK, Youk H. Autocrine Signaling and Quorum Sensing: Extreme Ends of a Common Spectrum. Trends Cell Biol. 2016;26(4):262–71. 10.1016/j.tcb.2015.11.002 26671200

[11] Ertao Z, Jianhui C, Chuangqi C, Changjiang Q, Sile C, Yulong H, et al Autocrine Sonic hedgehog signaling promotes gastric cancer proliferation through induction of phospholipase Cgamma1 and the ERK1/2 pathway. J Exp Clin Cancer Res. 2016;35(1):63. 27039174 10.1186/s13046-016-0336-9 PMC4818860

[12] Schlange T, Matsuda Y, Lienhard S, Huber A, Hynes NE. Autocrine WNT signaling contributes to breast cancer cell proliferation via the canonical WNT pathway and EGFR transactivation. Breast Cancer Res. 2007;9(5):R63 10.1186/bcr1769 17897439 PMC2242658

[13] Sporn MB, Todaro GJ. Autocrine secretion and malignant transformation of cells. N Engl J Med. 1980;303(15):878–80. 10.1056/NEJM198010093031511 7412807

[14] Woodhouse EC, Chuaqui RF, Liotta LA. General mechanisms of metastasis. Cancer. 1997;80(8 Suppl):1529–37. 9362419 10.1002/(sici)1097-0142(19971015)80:8+<1529::aid-cncr2>3.3.co;2-#

[15] Al-Hajj M, Wicha MS, Benito-Hernandez A, Morrison SJ, Clarke MF. Prospective identification of tumorigenic breast cancer cells. Proceedings of the National Academy of Sciences. 2003;100(7):3983–8. 10.1073/pnas.0530291100 PMC153034 12629218

[16] Brinster RL, Chen HY, Messing A, van Dyke T, Levine AJ, Palmiter RD. Transgenic mice harboring SV40 T-antigen genes develop characteristic brain tumors. Cell. 1984;37(2):367–79. 6327063 10.1016/0092-8674(84)90367-2 PMC4889224

[17] Cheon DJ, Orsulic S. Mouse models of cancer. Annu Rev Pathol. 2011;6:95–119. 10.1146/annurev.pathol.3.121806.154244 20936938

[18] Ambrose J, Livitz M, Wessels D, Kuhl S, Lusche DF, Scherer A, et al Mediated coalescence: a possible mechanism for tumor cellular heterogeneity. American journal of cancer research. 2015;5(11):3485–504. 26807328 PMC4697694

[19] Scherer A, Kuhl S, Wessels D, Lusche DF, Hanson B, Ambrose J, et al A Computer-Assisted 3D Model for Analyzing the Aggregation of Tumorigenic Cells Reveals Specialized Behaviors and Unique Cell Types that Facilitate Aggregate Coalescence. PLoS ONE. 2015;10(3):e0118628 10.1371/journal.pone.0118628 25790299 PMC4366230

[20] Slaughter DP, Southwick HW, Smejkal W. Field cancerization in oral stratified squamous epithelium; clinical implications of multicentric origin. Cancer. 1953;6(5):963–8. 13094644 10.1002/1097-0142(195309)6:5<963::aid-cncr2820060515>3.0.co;2-q

[21] Chu TY, Shen CY, Lee HS, Liu HS. Monoclonality and surface lesion-specific microsatellite alterations in premalignant and malignant neoplasia of uterine cervix: a local field effect of genomic instability and clonal evolution. Genes, chromosomes & cancer. 1999;24(2):127–34. 9885979 10.1002/(sici)1098-2264(199902)24:2<127::aid-gcc5>3.0.co;2-8

[22] Facista A, Nguyen H, Lewis C, Prasad AR, Ramsey L, Zaitlin B, et al Deficient expression of DNA repair enzymes in early progression to sporadic colon cancer. Genome integrity. 2012;3(1):3 10.1186/2041-9414-3-3 22494821 PMC3351028

[23] Franklin WA, Gazdar AF, Haney J, Wistuba II, La Rosa FG, Kennedy T, et al Widely dispersed p53 mutation in respiratory epithelium. A novel mechanism for field carcinogenesis. The Journal of clinical investigation. 1997;100(10):2417–639. 9329980 10.1172/JCI119748 PMC508406

[24] Heaphy CM, Griffith JK, Bisoffi M. Mammary field cancerization: molecular evidence and clinical importance. Breast cancer research and treatment. 2009;118(2):229–39. 10.1007/s10549-009-0504-0 19685287

[25] Jones TD, Wang M, Eble JN, MacLennan GT, Lopez-Beltran A, Zhang S, et al Molecular evidence supporting field effect in urothelial carcinogenesis. Clinical cancer research: an official journal of the American Association for Cancer Research. 2005;11(18):6512–9. 16166427 10.1158/1078-0432.CCR-05-0891

[26] Jothy S, Slesak B, Harlozinska A, Lapinska J, Adamiak J, Rabczynski J. Field effect of human colon carcinoma on normal mucosa: relevance of carcinoembryonic antigen expression. Tumour biology: the journal of the International Society for Oncodevelopmental Biology and Medicine. 1996;17(1):58–64. 7501974 10.1159/000217967

[27] Kadara H, Fujimoto J, Yoo SY, Maki Y, Gower AC, Kabbout M, et al Transcriptomic architecture of the adjacent airway field cancerization in non-small cell lung cancer. Journal of the National Cancer Institute. 2014;106(3):dju004 10.1093/jnci/dju004 24563515 PMC3982778

[28] Lochhead P, Chan AT, Nishihara R, Fuchs CS, Beck AH, Giovannucci E, et al Etiologic field effect: reappraisal of the field effect concept in cancer predisposition and progression. Modern pathology: an official journal of the United States and Canadian Academy of Pathology, Inc. 2014. 10.1038/modpathol.2014.81 PMC4265316 24925058

[29] Lynch SP, Lei X, Hsu L, Meric-Bernstam F, Buchholz TA, Zhang H, et al Breast cancer multifocality and multicentricity and locoregional recurrence. The oncologist. 2013;18(11):1167–73. 10.1634/theoncologist.2013-0167 24136008 PMC3825299

[30] Nonn L, Ananthanarayanan V, Gann PH. Evidence for field cancerization of the prostate. The Prostate. 2009;69(13):1470–9. 10.1002/pros.20983 19462462 PMC3690597

[31] Kuhl S, Voss E, Scherer A, Lusche DF, Wessels D, Soll DR. 4D Tumorigenesis model for quantitating coalescence, quantitating directed cell motility and chemotaxis, identifying unique cell behaviors and testing anti-cancer drugs In: Hereld D, Jin T, editors. Chemotaxis: Methods and Protocols: Springer; 2016. 10.1007/978-1-4939-3480-5_18 27271907

[32] Wessels D, Lusche DF, Kuhl S, Scherer A, Voss E, Soll DR. Quantitative Motion Analysis in Two and Three Dimensions. Methods Mol Biol. 2016;1365:265–92. 10.1007/978-1-4939-3124-8_14 26498790

[33] Dixit R, Cyr R. Cell damage and reactive oxygen species production induced by fluorescence microscopy: effect on mitosis and guidelines for non-invasive fluorescence microscopy. Plant J. 2003;36(2):280–90. 14535891 10.1046/j.1365-313x.2003.01868.x

[34] Jensen EC. Use of fluorescent probes: their effect on cell biology and limitations. Anat Rec (Hoboken). 2012;295(12):2031–6. 23060362 10.1002/ar.22602

[35] Sung MH. A checklist for successful quantitative live cell imaging in systems biology. Cells. 2013;2(2):284–93. 10.3390/cells2020284 24709701 PMC3972678

[36] Dobrucki JW, Feret D, Noatynska A. Scattering of exciting light by live cells in fluorescence confocal imaging: phototoxic effects and relevance for FRAP studies. Biophys J. 2007;93(5):1778–86. 10.1529/biophysj.106.096636 17416613 PMC1948059

[37] Pampaloni F, Berge U, Marmaras A, Horvath P, Kroschewski R, Stelzer EH. Tissue-culture light sheet fluorescence microscopy (TC-LSFM) allows long-term imaging of three-dimensional cell cultures under controlled conditions. Integr Biol (Camb). 2014;6(10):988–98. 25183478 10.1039/c4ib00121d

[38] Babapoor S, Horwich M, Wu R, Levinson S, Gandhi M, Makkar H, et al microRNA in situ hybridization for miR-211 detection as an ancillary test in melanoma diagnosis. Modern pathology: an official journal of the United States and Canadian Academy of Pathology, Inc. 2016;29(5):461–75. 10.1038/modpathol.2016.44 26916074

[39] Glasgow BJ, Wen DR, Al-Jitawi S, Cochran AJ. Antibody to S-100 protein aids the separation of pagetoid melanoma from mammary and extramammary Paget's disease. J Cutan Pathol. 1987;14(4):223–6. 2442212 10.1111/j.1600-0560.1987.tb01337.x

[40] Smoller BR. Histologic criteria for diagnosing primary cutaneous malignant melanoma. Modern pathology: an official journal of the United States and Canadian Academy of Pathology, Inc. 2006;19 Suppl 2:S34–40. 10.1038/modpathol.3800508 16446714

[41] Aung PP, Mutyambizi KK, Danialan R, Ivan D, Prieto VG. Differential diagnosis of heavily pigmented melanocytic lesions: challenges and diagnostic approach. J Clin Pathol. 2015;68(12):963–70. 10.1136/jclinpath-2015-202887 26602414

[42] Costa S, Byrne M, Pissaloux D, Haddad V, Paindavoine S, Thomas L, et al Melanomas Associated With Blue Nevi or Mimicking Cellular Blue Nevi: Clinical, Pathologic, and Molecular Study of 11 Cases Displaying a High Frequency of GNA11 Mutations, BAP1 Expression Loss, and a Predilection for the Scalp. Am J Surg Pathol. 2016;40(3):368–77. 10.1097/PAS.0000000000000568 26645730

[43] Guida S, Pellacani G, Cesinaro AM, Moscarella E, Argenziano G, Farnetani F, et al Spitz naevi and melanomas with similar dermoscopic patterns: can confocal microscopy differentiate? British Journal of Dermatology. 2016;174(3):610–6. 10.1111/bjd.14286 26554394

[44] Kutzner H, Metzler G, Argenyi Z, Requena L, Palmedo G, Mentzel T, et al Histological and genetic evidence for a variant of superficial spreading melanoma composed predominantly of large nests. Modern pathology: an official journal of the United States and Canadian Academy of Pathology, Inc. 2012;25(6):838–45. 10.1038/modpathol.2012.35 22388759

[45] Mihm MC Jr., Clark WH Jr., From L. The clinical diagnosis, classification and histogenetic concepts of the early stages of cutaneous malignant melanomas. N Engl J Med. 1971;284(19):1078–82. 10.1056/NEJM197105132841907 4929321

[46] Gupta PB, Kuperwasser C, Brunet J-P, Ramaswamy S, Kuo W-L, Gray JW, et al The melanocyte differentiation program predisposes to metastasis following neoplastic transformation. Nature genetics. 2005;37(10):1047–54. 10.1038/ng1634 16142232 PMC1694635

[47] Werb Z, Tremble PM, Behrendtsen O, Crowley E, Damsky CH. Signal transduction through the fibronectin receptor induces collagenase and stromelysin gene expression. J Cell Biol. 1989;109(2):877–89. 2547805 10.1083/jcb.109.2.877 PMC2115739

[48] Ahrens T, Sleeman JP, Schempp CM, Howells N, Hofmann M, Ponta H, et al Soluble CD44 inhibits melanoma tumor growth by blocking cell surface CD44 binding to hyaluronic acid. Oncogene. 2001;20(26):3399–408. 10.1038/sj.onc.1204435 11423990

[49] Gunthert U, Hofmann M, Rudy W, Reber S, Zoller M, Haussmann I, et al A new variant of glycoprotein CD44 confers metastatic potential to rat carcinoma cells. Cell. 1991;65(1):13–24. 1707342 10.1016/0092-8674(91)90403-l

[50] Herold-Mende C, Seiter S, Born AI, Patzelt E, Schupp M, Zoller J, et al Expression of CD44 splice variants in squamous epithelia and squamous cell carcinomas of the head and neck. J Pathol. 1996;179(1):66–73. 10.1002/(SICI)1096-9896(199605)179:1<66::AID-PATH544>3.0.CO;2-5 8691348

[51] Neri A, Welch D, Kawaguchi T, Nicolson GL. Development and biologic properties of malignant cell sublines and clones of a spontaneously metastasizing rat mammary adenocarcinoma. Journal of the National Cancer Institute. 1982;68(3):507–17. 6950180

[52] Raso-Barnett L, Banky B, Barbai T, Becsagh P, Timar J, Raso E. Demonstration of a melanoma-specific CD44 alternative splicing pattern that remains qualitatively stable, but shows quantitative changes during tumour progression. PLoS One. 2013;8(1):e53883 10.1371/journal.pone.0053883 23342032 PMC3544768

[53] Seiter S, Schadendorf D, Herrmann K, Schneider M, Rosel M, Arch R, et al Expression of CD44 variant isoforms in malignant melanoma. Clinical cancer research: an official journal of the American Association for Cancer Research. 1996;2(3):447–56. 9816190

[54] Seiter S, Tilgen W, Herrmann K, Schadendorf D, Patzelt E, Moller P, et al Expression of CD44 splice variants in human skin and epidermal tumours. Virchows Arch. 1996;428(3):141–9. 8688968 10.1007/BF00200656

[55] Fogh J, Wright WC, Loveless JD. Absence of HeLa cell contamination in 169 cell lines derived from human tumors. Journal of the National Cancer Institute. 1977;58(2):209–14. 833871 10.1093/jnci/58.2.209

[56] Moore GE, Gerner RE. Malignant melanoma. Surg Gynecol Obstet. 1971;132(3):427–36. 5546295

[57] Clark EA, Golub TR, Lander ES, Hynes RO. Genomic analysis of metastasis reveals an essential role for RhoC. Nature. 2000;406(6795):532–5. 10.1038/35020106 10952316

[58] Russ JC. The Image Processing Handbook. Boca Raton, Florida: CRC Press, Inc.; 1992.

[59] Soll D, Voss E. Two and three dimensional computer systems for analyzing how cells crawl In: Soll D, Wessels D, editors. Motion Analysis of Living Cells: John Wiley, Inc 1998 p. 25–52.

[60] Soll DR. The use of computers in understanding how animal cells crawl. Int Rev Cytol. 1995;163:43–104. 8522423

[61] Soll DR. Computer-assisted three-dimensional reconstruction and motion analysis of living, crawling cells. Comput Med Imaging Graph. 1999;23(1):3–14. 10091863 10.1016/s0895-6111(98)00058-5

[62] Soll DR, Voss E, Johnson O, Wessels D. Three-dimensional reconstruction and motion analysis of living, crawling cells. Scanning. 2000;22(4):249–57. 10958392 10.1002/sca.4950220404

[63] Soll DR, Wessels D, Heid PJ, Voss E. Computer-assisted reconstruction and motion analysis of the three-dimensional cell. Scientific World Journal. 2003;3(Journal Article):827–41. 10.1100/tsw.2003.70 14532423 PMC5974884

[64] Soll DR, Wessels D, Kuhl S, Lusche DF. How a cell crawls and the role of cortical myosin II. Eukaryot Cell. 2009;8(9):1381–96. 10.1128/EC.00121-09 19633268 PMC2747829

[65] Soll DR, Wessels D, Voss E, Johnson O. Computer-assisted systems for the analysis of amoeboid cell motility. Methods Mol Biol. 2001;161:45–58. 11190516 10.1385/1-59259-051-9:045

[66] Wessels D, Vawter-Hugart H, Murray J, Soll DR. Three-dimensional dynamics of pseudopod formation and the regulation of turning during the motility cycle of Dictyostelium. Cell Motil Cytoskel. 1994;27(1):1–12. 10.1002/cm.970270102 8194106

[67] Poston T, Wong TT, Heng PA. Multiresolution isosurface extraction with the adaptive skeleton climbing. Computer Graphics Forum. 1998;17:137–47.

[68] Lorensen WE, Cline HE. Marching cubes: A high resolution 3D surface construction algorithm. ACM SIGGRAPH Computer Graphics. 1987;21:163–9.

[69] Hermann L. Laplacian-isoparametric grid generation scheme. J Engin Mech Div. 1976;102:749–56.

[70] Boyd DD, Kim SJ, Wang H, Jones TR, Gallick GE. A urokinase-derived peptide (A6) increases survival of mice bearing orthotopically grown prostate cancer and reduces lymph node metastasis. Am J Pathol. 2003;162(2):619–26. 10.1016/S0002-9440(10)63855-2 12547719 PMC1851141

[71] Cabezon T, Gromova I, Gromov P, Serizawa R, Timmermans Wielenga V, Kroman N, et al Proteomic profiling of triple-negative breast carcinomas in combination with a three-tier orthogonal technology approach identifies Mage-A4 as potential therapeutic target in estrogen receptor negative breast cancer. Mol Cell Proteomics. 2013;12(2):381–94. 10.1074/mcp.M112.019786 23172894 PMC3567861

[72] Li X, Hughes SC, Wevrick R. Evaluation of melanoma antigen (MAGE) gene expression in human cancers using The Cancer Genome Atlas. Cancer Genet. 2015;208(1–2):25–34. 10.1016/j.cancergen.2014.11.005 25592766

[73] Wong PP, Yeoh CC, Ahmad AS, Chelala C, Gillett C, Speirs V, et al Identification of MAGEA antigens as causal players in the development of tamoxifen-resistant breast cancer. Oncogene. 2014;33(37):4579–88. 10.1038/onc.2014.45 24662835 PMC4162461

[74] Blessing K, Sanders DS, Grant JJ. Comparison of immunohistochemical staining of the novel antibody melan-A with S100 protein and HMB-45 in malignant melanoma and melanoma variants. Histopathology. 1998;32(2):139–46. 9543670 10.1046/j.1365-2559.1998.00312.x

[75] Nonaka D, Chiriboga L, Rubin BP. Differential expression of S100 protein subtypes in malignant melanoma, and benign and malignant peripheral nerve sheath tumors. J Cutan Pathol. 2008;35(11):1014–9. 10.1111/j.1600-0560.2007.00953.x 18547346

[76] Gown AM, Vogel AM, Hoak D, Gough F, McNutt MA. Monoclonal antibodies specific for melanocytic tumors distinguish subpopulations of melanocytes. Am J Pathol. 1986;123(2):195–203. 3518473 PMC1888307

[77] Saalbach A, Kraft R, Herrmann K, Haustein UF, Anderegg U. The monoclonal antibody AS02 recognizes a protein on human fibroblasts being highly homologous to Thy-1. Arch Dermatol Res. 1998;290(7):360–6. 9749990 10.1007/s004030050318

[78] Im S, Hann SK, Park YK, Kim HI. Culture of melanocytes obtained from normal and vitiligo subjects. Yonsei Med J. 1992;33(4):344–50. 10.3349/ymj.1992.33.4.344 1309014

[79] Jungbauer S, Kemkemer R, Gruler H, Kaufmann D, Spatz JP. Cell shape normalization, dendrite orientation, and melanin production of normal and genetically altered (haploinsufficient NF1)-melanocytes by microstructured substrate interactions. Chemphyschem. 2004;5(1):85–92. 10.1002/cphc.200300868 14999847

[80] Provance DW Jr., Wei M, Ipe V, Mercer JA. Cultured melanocytes from dilute mutant mice exhibit dendritic morphology and altered melanosome distribution. Proc Natl Acad Sci U S A. 1996;93(25):14554–8. 8962090 10.1073/pnas.93.25.14554 PMC26171

[81] Giard DJ, Aaronson SA, Todaro GJ, Arnstein P, Kersey JH, Dosik H, et al In vitro cultivation of human tumors: establishment of cell lines derived from a series of solid tumors. Journal of the National Cancer Institute. 1973;51(5):1417–23. 4357758 10.1093/jnci/51.5.1417

[82] Kozlowski JM, Hart IR, Fidler IJ, Hanna N. A human melanoma line heterogeneous with respect to metastatic capacity in athymic nude mice. Journal of the National Cancer Institute. 1984;72(4):913–7. 6584666

[83] Goodfellow PN, Banting G, Wiles MV, Tunnacliffe A, Parkar M, Solomon E, et al The gene, MIC4, which controls expression of the antigen defined by monoclonal antibody F10.44.2, is on human chromosome 11. Eur J Immunol. 1982;12(8):659–63. 10.1002/eji.1830120807 7140811

[84] Piotrowicz RS, Damaj BB, Hachicha M, Incardona F, Howell SB, Finlayson M. A6 Peptide Activates CD44 Adhesive Activity, Induces FAK and MEK Phosphorylation, and Inhibits the Migration and Metastasis of CD44-Expressing Cells. Molecular Cancer Therapeutics. 2011;10(11):2072–82. 10.1158/1535-7163.MCT-11-0351 21885863

[85] Teriete P, Banerji S, Noble M, Blundell CD, Wright AJ, Pickford AR, et al Structure of the regulatory hyaluronan binding domain in the inflammatory leukocyte homing receptor CD44. Mol Cell. 2004;13(4):483–96. 14992719 10.1016/s1097-2765(04)00080-2

[86] Kirfel G, Herzog V. Migration of epidermal keratinocytes: mechanisms, regulation, and biological significance. Protoplasma. 2004;223(2–4):67–78. 10.1007/s00709-003-0031-5 15221512

[87] Chen Y, Mistry DS, Sen GL. Highly rapid and efficient conversion of human fibroblasts to keratinocyte like cells. The Journal of investigative dermatology. 2014;134(2):335–44. 10.1038/jid.2013.327 23921950 PMC3875612

[88] Song PI, Neparidze N, Armstrong CA, Ansel JC, Park Y-M, Abraham T, et al Human Keratinocytes Express Functional CD14 and Toll-Like Receptor 4. Journal of Investigative Dermatology. 2002;119(2):424–32. 10.1046/j.1523-1747.2002.01847.x 12190866

[89] Kerosuo L, Bronner-Fraser M. What is bad in cancer is good in the embryo: importance of EMT in neural crest development. Seminars in cell & developmental biology. 2012;23(3):320–32. 22430756 10.1016/j.semcdb.2012.03.010 PMC3345076

[90] Bronner-Fraser M, Sieber-Blum M, Cohen AM. Clonal analysis of the avian neural crest: migration and maturation of mixed neural crest clones injected into host chicken embryos. The Journal of comparative neurology. 1980;193(2):423–34. 10.1002/cne.901930209 7440776

[91] Ezin AM, Fraser SE, Bronner-Fraser M. Fate map and morphogenesis of presumptive neural crest and dorsal neural tube. Developmental biology. 2009;330(2):221–36. 10.1016/j.ydbio.2009.03.018 19332051 PMC2717095

[92] Zambruno G, Marchisio PC, Melchiori A, Bondanza S, Cancedda R, De Luca M. Expression of integrin receptors and their role in adhesion, spreading and migration of normal human melanocytes. J Cell Sci. 1993;105 (Pt 1):179–90. 8360272 10.1242/jcs.105.1.179

[93] Leotlela PD, Wade MS, Duray PH, Rhode MJ, Brown HF, Rosenthal DT, et al Claudin-1 overexpression in melanoma is regulated by PKC and contributes to melanoma cell motility. Oncogene. 2007;26(26):3846–56. 10.1038/sj.onc.1210155 17160014

[94] Wang YG, Kim SJ, Baek JH, Lee HW, Jeong SY, Chun KH. Galectin-3 increases the motility of mouse melanoma cells by regulating matrix metalloproteinase-1 expression. Exp Mol Med. 2012;44(6):387–93. 10.3858/emm.2012.44.6.044 22437631 PMC3389077

[95] Weeraratna AT, Jiang Y, Hostetter G, Rosenblatt K, Duray P, Bittner M, et al Wnt5a signaling directly affects cell motility and invasion of metastatic melanoma. Cancer Cell. 2002;1(3):279–88. 12086864 10.1016/s1535-6108(02)00045-4

[96] Gov NS. Collective cell migration patterns: follow the leader. Proc Natl Acad Sci U S A. 2007;104(41):15970–1. 10.1073/pnas.0708037104 17913874 PMC2042145

[97] Omelchenko T, Vasiliev JM, Gelfand IM, Feder HH, Bonder EM. Rho-dependent formation of epithelial "leader" cells during wound healing. Proc Natl Acad Sci U S A. 2003;100(19):10788–93. 10.1073/pnas.1834401100 12960404 PMC196881

[98] Poujade M, Grasland-Mongrain E, Hertzog A, Jouanneau J, Chavrier P, Ladoux B, et al Collective migration of an epithelial monolayer in response to a model wound. Proc Natl Acad Sci U S A. 2007;104(41):15988–93. 10.1073/pnas.0705062104 17905871 PMC2042149

[99] Stoker M, Gherardi E, Perryman M, Gray J. Scatter factor is a fibroblast-derived modulator of epithelial cell mobility. Nature. 1987;327(6119):239–42. 10.1038/327239a0 2952888

[100] Weaver VM, Petersen OW, Wang F, Larabell CA, Briand P, Damsky C, et al Reversion of the malignant phenotype of human breast cells in three-dimensional culture and in vivo by integrin blocking antibodies. J Cell Biol. 1997;137(1):231–45. 9105051 10.1083/jcb.137.1.231 PMC2139858

[101] Baugher PJ, Krishnamoorthy L, Price JE, Dharmawardhane SF. Rac1 and Rac3 isoform activation is involved in the invasive and metastatic phenotype of human breast cancer cells. Breast Cancer Res. 2005;7(6):R965–74. 10.1186/bcr1329 16280046 PMC1410764

[102] Price JE, Polyzos A, Zhang RD, Daniels LM. Tumorigenicity and metastasis of human breast carcinoma cell lines in nude mice. Cancer Res. 1990;50(3):717–21. 2297709

[103] Desgrosellier JS, Cheresh DA. Integrins in cancer: biological implications and therapeutic opportunities. Nat Rev Cancer. 2010;10(1):9–22. 10.1038/nrc2748 20029421 PMC4383089

[104] Ley K, Rivera-Nieves J, Sandborn WJ, Shattil S. Integrin-based therapeutics: biological basis, clinical use and new drugs. Nat Rev Drug Discov. 2016;15(3):173–83. 10.1038/nrd.2015.10 26822833 PMC4890615

[105] Fridman R, Kibbey MC, Royce LS, Zain M, Sweeney M, Jicha DL, et al Enhanced tumor growth of both primary and established human and murine tumor cells in athymic mice after coinjection with Matrigel. Journal of the National Cancer Institute. 1991;83(11):769–74. 1789823 10.1093/jnci/83.11.769

[106] Kibbey MC. Maintenance of the EHS sarcoma and Matrigel preparation. Journal of Tissue Culture Methods. 1994;16:227–30.

[107] Sixma JJ, van Zanten GH, Huizinga EG, van der Plas RM, Verkley M, Wu YP, et al Platelet adhesion to collagen: an update. Thromb Haemost. 1997;78(1):434–8. 9198192

[108] Sweeney TM, Kibbey MC, Zain M, Fridman R, Kleinman HK. Basement membrane and the SIKVAV laminin-derived peptide promote tumor growth and metastases. Cancer Metastasis Rev. 1991;10(3):245–54. 1764767 10.1007/BF00050795

[109] Moody G, Belmontes B, Masterman S, Wang W, King C, Murawsky C, et al Antibody-mediated neutralization of autocrine Gas6 inhibits the growth of pancreatic ductal adenocarcinoma tumors in vivo. Int J Cancer. 2016;139(6):1340–9. 10.1002/ijc.30180 27170265

[110] Guo Y, Ma J, Wang J, Che X, Narula J, Bigby M, et al Inhibition of human melanoma growth and metastasis in vivo by anti-CD44 monoclonal antibody. Cancer Res. 1994;54(6):1561–5. 7511044

[111] Siiskonen H, Poukka M, Tyynela-Korhonen K, Sironen R, Pasonen-Seppanen S. Inverse expression of hyaluronidase 2 and hyaluronan synthases 1–3 is associated with reduced hyaluronan content in malignant cutaneous melanoma. BMC Cancer. 2013;13:181 10.1186/1471-2407-13-181 23560496 PMC3626669

[112] Sironen RK, Tammi M, Tammi R, Auvinen PK, Anttila M, Kosma VM. Hyaluronan in human malignancies. Exp Cell Res. 2011;317(4):383–91. 10.1016/j.yexcr.2010.11.017 21134368

[113] Ponta H, Sherman L, Herrlich PA. CD44: from adhesion molecules to signalling regulators. Nat Rev Mol Cell Biol. 2003;4(1):33–45. 10.1038/nrm1004 12511867

[114] Naor D, Sionov RV, Ish-Shalom D. CD44: structure, function, and association with the malignant process. Adv Cancer Res. 1997;71:241–319. 9111868 10.1016/s0065-230x(08)60101-3

[115] Yu Q, Stamenkovic I. Localization of matrix metalloproteinase 9 to the cell surface provides a mechanism for CD44-mediated tumor invasion. Genes Dev. 1999;13(1):35–48. 9887098 10.1101/gad.13.1.35 PMC316376

[116] Belitsos PC, Hildreth JE, August JT. Homotypic cell aggregation induced by anti-CD44(Pgp-1) monoclonal antibodies and related to CD44(Pgp-1) expression. J Immunol. 1990;144(5):1661–70. 1689752

[117] Ma Q, Zhou Y, Ma B, Chen X, Wen Y, Liu Y, et al The clinical value of CXCR4, HER2 and CD44 in human osteosarcoma: A pilot study. Oncol Lett. 2012;3(4):797–801. 10.3892/ol.2012.558 22740996 PMC3362375

