# Supplementary figures and images for: Melanoma cells undergo aggressive coalescence in a 3D Matrigel model that is repressed by anti-CD44
Source: PLoS One. 2017 Mar 6;12(3):e0173400. doi: 10.1371/journal.pone.0173400 (PMC5338862; doi:10.1371/journal.pone.0173400)

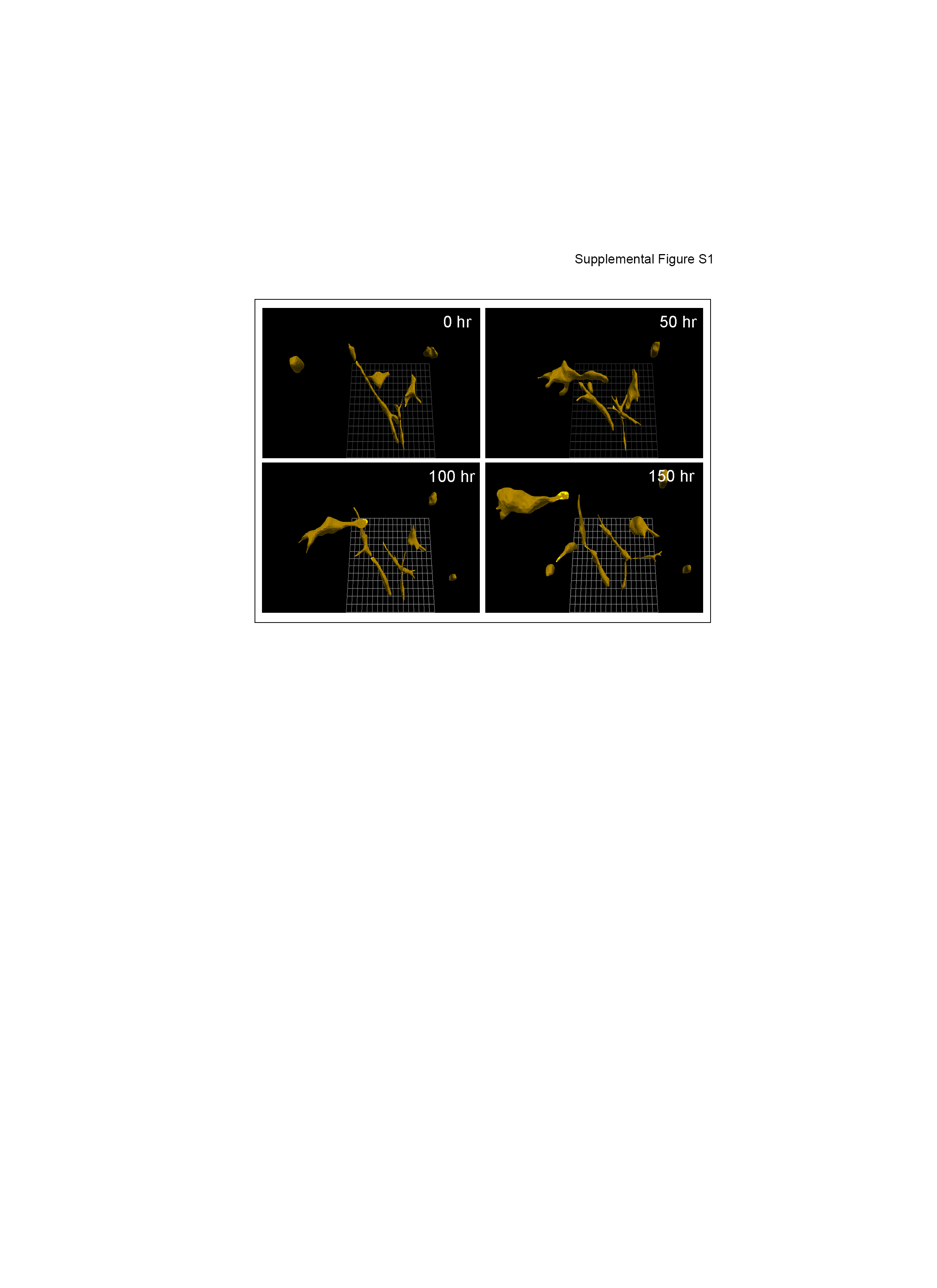

Supplement: S1 Fig — These cells exhibit the same dendritic morphology and rapid motility as melanocytes from normal skin tissue and, like melanocytes from normal skin, do not coalesce. (TIF) [file pone.0173400.s001.tif]

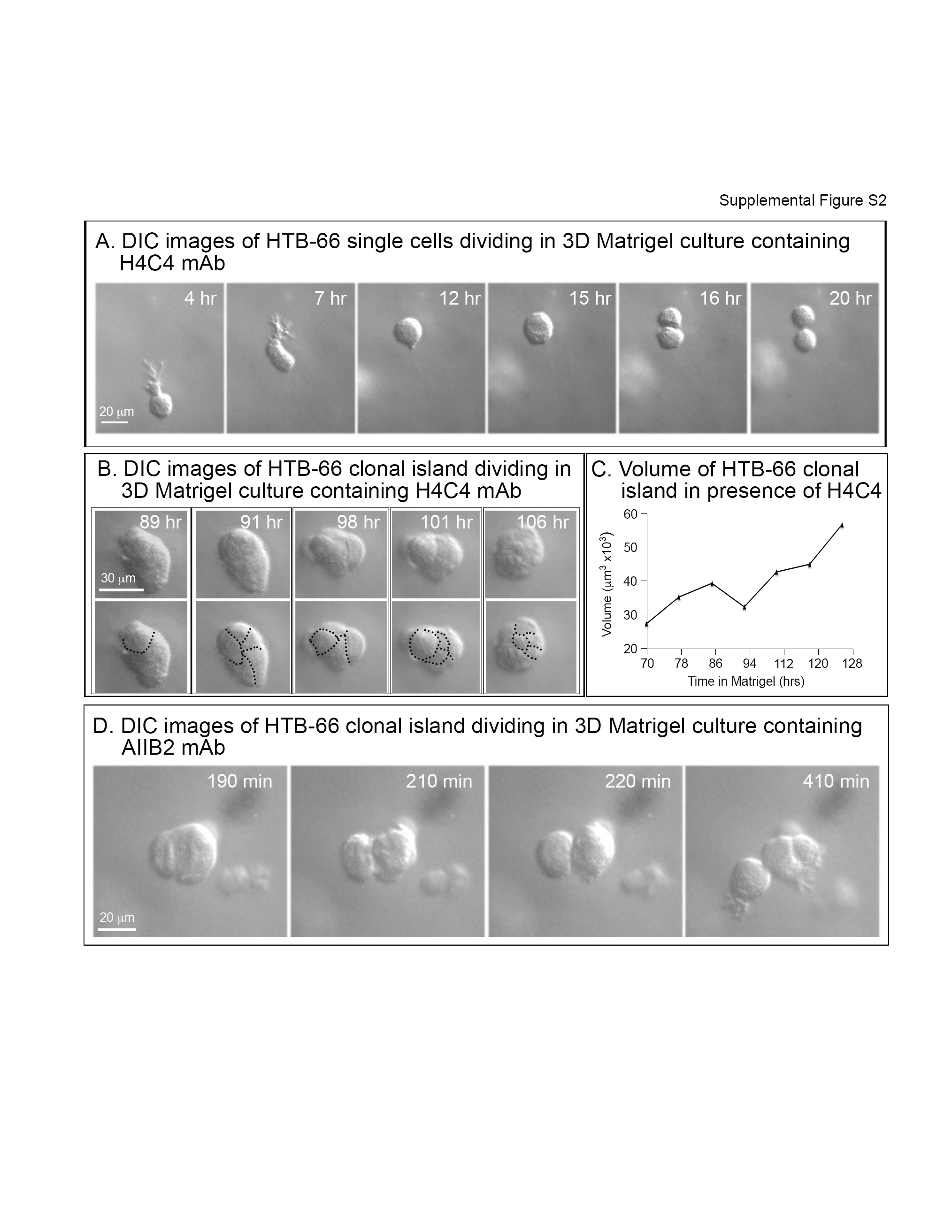

Supplement: S2 Fig — A. A representative time sequence of DIC images of a single cell taken at one depth in a 3D Matrigel culture in the presence of the H4C4 mAb reveals cell growth, division and cytokinesis by 20 hours. Cell division occurred in a majority of cells in these preparations. B. Cell division is visible in a clonal island of HTB-66 cells taken at one depth in a 3D Matrigel culture in the presence of the H4C4 mAb. Dotted black lines in the second row highlight the cleavage furrows at the time points given in the upper row. C. J3D-DIAS4.2 calculations of the volume increase over time of the clonal island in B support the conclusion that cell division is continuing in the presence of the H4C4 mAb. D. DIC images of a single cell taken at one depth in a 3D Matrigel culture of HTB-66 cells in the presence of the AIIB2 mAb reveal cell division. Scale bars are in the lower left of the first panel in each DIC series. (TIF) [file pone.0173400.s002.tif]

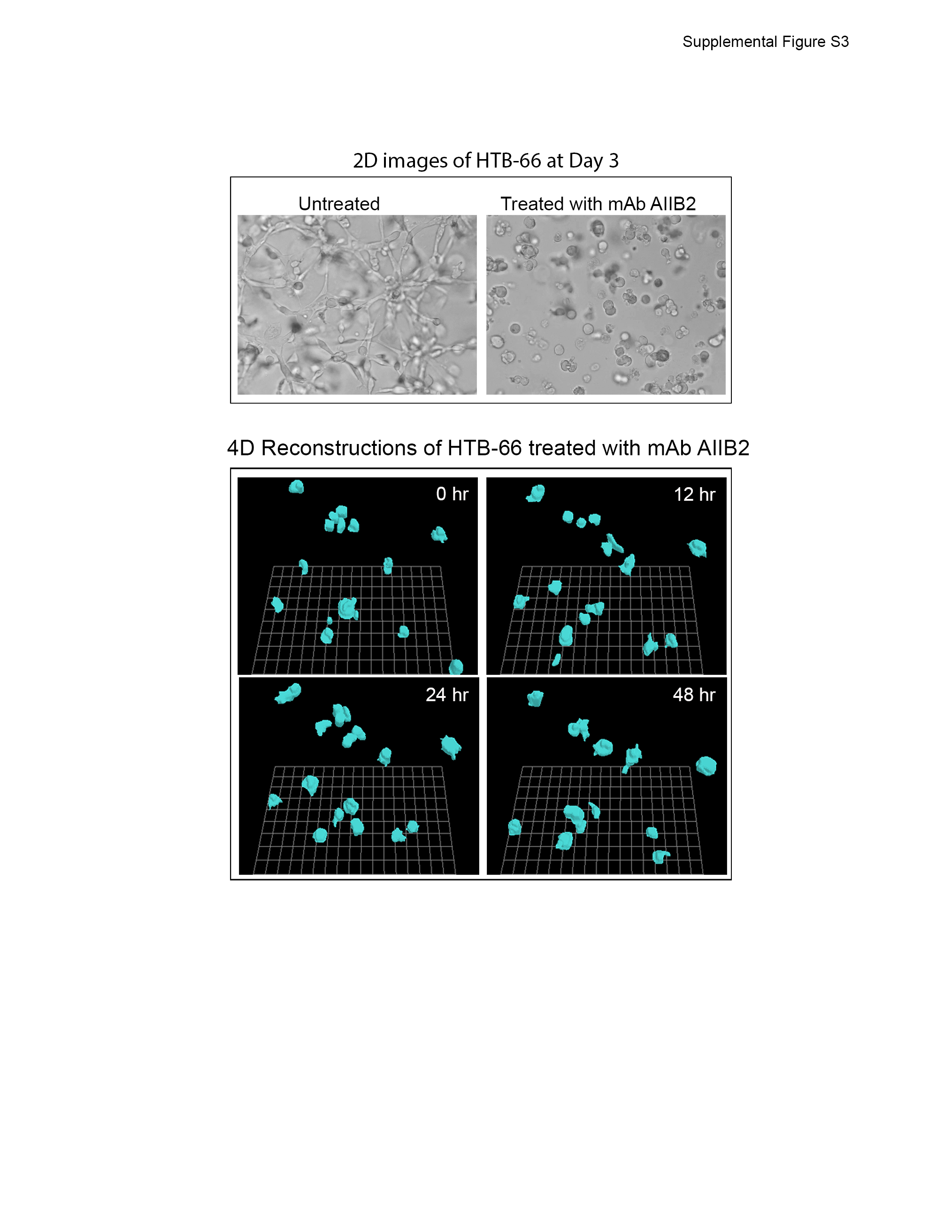

Supplement: S3 Fig — A. Brightfield images of untreated and AIIB2 treated HTB-66 cells in the 2D screen show that coalescence is inhibited through Day 3. B. J3D-DIAS4.2 reconstructions of HTB-66 cells in the 3D Matrigel culture over a 48 hour period in the presence of the mAb AIIB2 reveal that coalescence is inhibited. (TIF) [file pone.0173400.s003.tif]
