# Supplementary material for: Melanoma cells undergo aggressive coalescence in a 3D Matrigel model that is repressed by anti-CD44
Source: PLoS One. 2017 Mar 6;12(3):e0173400. doi: 10.1371/journal.pone.0173400 (PMC5338862; doi:10.1371/journal.pone.0173400)
Supplement: S1 Table — (PDF) [file pone.0173400.s005.pdf]

S1 Table. mAbs used to stain cells for melanoma phenotype.

			Antibody Characterization									
Antibody	Antigen	Antigen specimen	WB	IP	IHC	IF	FACS	ELISA	FB	FFPE	Epitope	MA
CPTC-MageA4-3 (DSHB)	Melanoma Antigen Family A, 4	human	+			t.s.		+				
CPTC-S100A4-1 (DSHB)	S100 calcium binding protein A2	human	+			t.s.		+				
CP28 (Calbiochem(AS02)	anti-fibroblast/ CD90/Thy-1	human	+	+	+	+	+					
HMB45 (Dako)	Pme117	human	-		+	+						

*ts, this study
