# Supplementary material for: Melanoma cells undergo aggressive coalescence in a 3D Matrigel model that is repressed by anti-CD44
Source: PLoS One. 2017 Mar 6;12(3):e0173400. doi: 10.1371/journal.pone.0173400 (PMC5338862; doi:10.1371/journal.pone.0173400)
Supplement: S2 Table — (PDF) [file pone.0173400.s006.pdf]

S2 Table. mAbs from DSHB used to screen for inhibition of coalescence.

				Antibody Characterization									
**DSHB Antibody**	**Antigen**	**Antigen specimen**	**Melanoma cell coalescence (+ or -)**	**WB**	**IP**	**IHC**	**IF**	**FACS**	**ELISA**	**FB**	**FFPE**	**Epitope**	**MA**
AIIB2 purified	Integrin, beta-1 subunit (CD29)	human	+	+	+	+	+	+	+	+	+		
ANTI-GRL(1) purified	Leukocyte cell surface glycoprotein	chicken no information on test on human	-		+								
AON-1 purified	Osteonectin	human	-	+	+	+							
BIIG2 purified	Integrin alpha-5	human	-		+	+		+		+			
B30 purified	neuronal, mesencephalic trigeminal cell surface marker	mouse, rat no information on test on human	-	+		+							
B2C11 purified	CD9	rat not tested on human yet	-							+			
CPTC-PTEN-1 purified	PTEN	human	-	+		+							
CPTC-PTEN-2 purified	PTEN	human	-	+		+							
CPTC-EGFR-1 purified	EGFR	human	-						+				
CPTC-IL-6-1 purified	Interleukin 6	human	-	+					+				
CPTC-TNFRSF9-3 purified	Tumor necrosis factor receptor superfamily member 9	human	-	+					+				
CPTC-TACSTD2-2 purified	Tumor-associated calcium signal transducer 2	human	-	+					+				
CPTC-TACSTD2-3 purified	Tumor-associated calcium signal transducer 2	human	-	+					+				
CSAT purified	integrin beta-1	avian no information on test on human	-	+						+			
D71E2 purified	integrin alpha-5	avian no information on test on human	**slight**	+									

EMA-1 supernatant	Primordial germ cell surface marker	human	-	+		+						
F461.3B purified	Notch, extracellular domain, EGF repeats #5-7	Drosophila no information on test on human	-	+			+					
G1/221/12 purified	transferrin receptor	human	-	+	+	+						
HB#7 purified	CD133/ Prominin-1	human	-	+		+	+	+				
H36 purified	p46 cell surface protein excluded from macropinocytic cup	D.discoideum no information on test on human	-	+	+	+						
H4C4 purified	CD44	human	+	+			ts	+	+	ts		
H5A4 purified	CD11b	human	-									
H5C6 : H5C6-s purified	CD63 (human) LAMP	human	-	+								
MAb83F6 supernatant	TRP protein (transient receptor potential)	Drosophila no information on test on human	-	+		+						+
MAb83F6 purified	TRP protein (transient receptor potential)	Drosophila no information on test on human	-	+		+						+
MRN2 2B7 purified	mineralocorticoid receptor	rat no information on test on human	-	+		+						
MRN3 3F10 purified	mineralocorticoid receptor	rat no information on test on human	-			+						
MC-480 supernatant	SSEA-1/ alpha-(1,3)-fucosyltransferase / CD15	human	-	+	+	+	+	+			+	
MC-480 purified	SSEA-1/ alpha-(1,3)-fucosyltransferase / CD15	human	-	+	+	+	+	+			+	
MC-631 purified	SSEA-3	human	-			+						
MC-813 supernatant	SSEA-4	human	-	+		+	+	+	+			
MHM.24 purified	CD11a (LFA-1, alpha subunit),human	human	-	+	+	+				+		+

MPIIIB10(1) purified	osteopontin	human	-	+		+							
PCRP-ESRRA-1B10 purified	PCRP-ESRRA-1B10	human	-	+	+								+
PCRP-ESRRG-3A6 purified	ESRRG; Estrogen-related receptor gamma	human	-		+								+
PCRP-ING1-1C2	Inhibitor of growth family, member 1	human	-	+									+
PCRP-RORA-2C2 purified	RORA; RAR-related orphan receptor A	human	-		+								+
PCRP-RXRA-2A8 purified	RXRA; Retinoid X receptor, alpha	human	-		+								+
PCRP-RXRG-5C9 purified	Retinoid X receptor, gamma	human	-		+								+
P1E6	integrin alpha-2	human	-	+	+	+		+			+	+	
P1F6 purified	integrin alpha V/5 + beta 5 (CD49e, CD51, fibronectin/vitronectin receptor)	human	-		+	+			+	+			
P3H9	laminin 5	human	-	+	+			+	+			+	
P4H9	integrin beta-2 (CD18)	human	-		+	+		+			+		
P2C62C4 purified	integrin alpha-6 (avian)		**slight**	+		+							
QH 1 purified	Quail endothelial cell surface	Quail no information on test on human	-			+	+						
rMR1-18 1D5n purified	mineralocorticoid receptor	human	-	+	+	+							
SM 1.2 purified	somatomedin-C; Sm-C/IGF-1	human	-	+		+				+		+	
STRO-1 purified	stromal cell surface marker	human	-				+	+					
VD2(1)B12 purified	DHPR beta subunit, skeletal muscle	human	-	+	+	+							
151-IgG purified	Epidermal growth factor receptor	rat, no information on test on human	-	+	+								
1D10 purified	CD44	chicken, no information on test on human	-	+	+	+							

5.1H11 purified	NCAM	human	-				+		
7D6 purified	L-CAM	chicken, does not react with mammals	-		+	+			
8.1.1 purified	Podoplanin	mouse, no information on test on human	-	+		+		+	
8B4D2 MH2B purified	Glutamate receptor subunit, DGluR-IIA	Drosophila, no information on test on human	-	+		+	+		
8B22F5 purified	protein tyrosine phosphatase, receptor-linked, DPTP10D	Drosophila, no information on test on human	-	+	+	+			
8H9 purified	CD64/FcgRI	human	-						+
